# Glycan microarray analysis of *Candida*-related antibodies in human and mice sera guides biomarker discovery and vaccine development

**DOI:** 10.1073/pnas.2505340122

**Published:** 2025-09-25

**Authors:** Emelie E. Reuber, Emer Hickey, Arnab Pradhan, Rosanne Sprute, Tilman Lingscheid, Pinkus Tober-Lau, Ian Leaves, Mark H. T. Stappers, Florian Kurth, Mariolina Bruno, Mihai G. Netea, Leif E. Sander, Oliver A. Cornely, Neil A. R. Gow, Alistair J. P. Brown, Rajat K. Singh, Sabrina Omoregbee-Leichnitz, Eric T. Sletten, José Danglad-Flores, Peter H. Seeberger

**Affiliations:** ^a^Institute of Chemistry and Biochemistry, Freie Universität Berlin, Berlin 14195, Germany; ^b^Biomolecular Systems Department, Max Planck Institute of Colloids and Interfaces, Potsdam 14476, Germany; ^c^Medical Research Council Centre for Medical Mycology at the University of Exeter, University of Exeter, Exeter EX4 4QD, United Kingdom; ^d^Institute of Translational Research, Faculty of Medicine and University Hospital Cologne, Cologne Excellence Cluster on Cellular Stress Responses in Aging-Associated Diseases, University of Cologne, Cologne 50931, Germany; ^e^Faculty of Medicine and University Hospital Cologne, Department I of Internal Medicine, Center for Integrated Oncology Aachen Bonn Cologne Duesseldorf and Excellence Center for Medical Mycology, University of Cologne, Cologne 50931, Germany; ^f^German Centre for Infection Research, Partner Site Bonn-Cologne, Cologne 50931, Germany; ^g^Department of Infectious Diseases and Critical Care Medicine, Campus Virchow-Klinikum and Campus Charité Mitte, Charité—Universitätsmedizin Berlin, Corporate Member of Freie Universität and Humboldt-Universität zu Berlin, Berlin 10117, Germany; ^h^Centre de Recherches Médicales de Lambaréné, Lambaréné 242, Gabon; ^i^German Center for Lung Research, Berlin 10115, Germany; ^j^Department of Internal Medicine and Radboud Center for Infectious Diseases, Radboud University Medical Center, Nijmegen 6525, The Netherlands; ^k^Department of Immunology and Metabolism, Life and Medical Sciences Institute, University of Bonn, Bonn 53127, Germany; ^l^Berlin Institute of Health at Charité—Universitätsmedizin Berlin, Berlin 10178, Germany; ^m^Clinical Trials Centre Cologne, Faculty of Medicine and University Hospital Cologne, University of Cologne, Cologne 50931, Germany

**Keywords:** *Candida*, glycan, microarray, antibody

## Abstract

Invasive, high-mortality yeast infections caused by pathogenic *Candida* species are the most common fungal nosocomial bloodstream infections. The World Health Organization (WHO) has called for improved prevention and diagnostic strategies for fungal pathogens. Here, we demonstrate that *Candida*-related antibodies can be detected using glycan microarrays containing synthetic glycans to guide the discovery of oligosaccharide epitopes for diagnostics and vaccine design. Pure, synthetic mannans and *β*-glucans, resembling carbohydrates found on the surface of *Candida* spp., were employed to screen and compare sera from infected humans and mice with noninfected individuals. IgM antibodies directed toward *β*-glucans were observed shortly after infection, and after a longer time of infection, IgM and IgG antibodies that preferentially recognize mannans. Phosphodiester-linked mannosides and the *β*-(1,2)-mannose monomer help to distinguish different *Candida* spp. The tetrasaccharide antigen *β*-(1,2)Man-*α*-(1,2)Man-*α*-(1,2)Man-*α*-(1,2)Man, and the pentasaccharide antigens *α*-(1,2)Man-*α*-(1,3)Man-*α*-(1,2)Man-*α*-(1,2)Man-*α*-(1,2)Man and *β*-(1,3)Glc-*β*-(1,3)Glc-*β*-(1,3)Glc-[*β*-(1,6)Glc]-*β*-(1,3)Glc were identified as potential epitopes for detection, e.g., through monoclonal antibody lateral flow tests, and the development of glycoconjugate or monoclonal antibody vaccines against *Candida.*

## Significance

Insights into the immune response following yeast infections with *Candida* provide a basis for developing preventative and diagnostic strategies. Microarrays containing synthetic glycans resembling carbohydrates found on the surface of *Candida* helped us to detect IgG and IgM antibodies in sera from infected mice and humans. While IgM antibodies are initially directed against *β*-glucans, later IgM and IgG antibodies recognize predominantly oligomannoses. Different *Candida* spp. can be distinguished using *β*-(1,2)-mannose monomer which could be employed in a diagnostic setting. Three oligosaccharides, *β*-(1,2)Man-*α*-(1,2)Man-*α*-(1,2)Man-*α*-(1,2)Man, *α*-(1,2)Man-*α*-(1,3)Man-*α*-(1,2)Man-*α*-(1,2)Man-*α*-(1,2)Man, and *β*-(1,3)Glc-*β*-(1,3)Glc-*β*-(1,3)Glc-[*β*-(1,6)Glc]-*β*-(1,3)Glc, emerged as leads for the development of diagnostics and vaccines against *Candida* spp.

Invasive infections by yeasts of the genus *Candida*, including bloodstream infection (candidemia), affect over 1.5 million people worldwide every year, resulting in about one million deaths, and are the fourth most common nosocomial bloodstream infection among intensive care unit patients (1, 2). *Candida* are phylogenetically diverse groups of species, some of which have been reclassified into separate genera, and the traditional nomenclature will be used (3–5). Five *Candida* species are responsible for more than 90% of invasive candidiasis cases, ranked from the most to the least common: *Candida albicans*, *Candida glabrata (Nakaseomyces glabratus), Candida parapsilosis*, *Candida tropicalis*, and *Candida krusei* (*Pichia kudriavzevii*) (6). Recent major outbreaks of invasive candidiasis (IC) around the world are linked to the multidrug-resistant *C. auris (Candidozyma auris)* (7–9). The World Health Organization’s (WHO) first priority group ranking of fungal pathogens listed *C. albicans* and *C. auris* as “critical priority pathogens” and *C. glabrata*, *C. tropicalis*, and *C. parapsilosis* as “high priority pathogens” in 2022. Late diagnosis of invasive candidiasis delays initiation of effective treatment and leads to a poor prognosis (10). A better understanding of virulence mechanisms, immune escape, antifungal resistance, and disease burden is needed to improve diagnostic and prevention strategies (11).

**Fig. 1.**
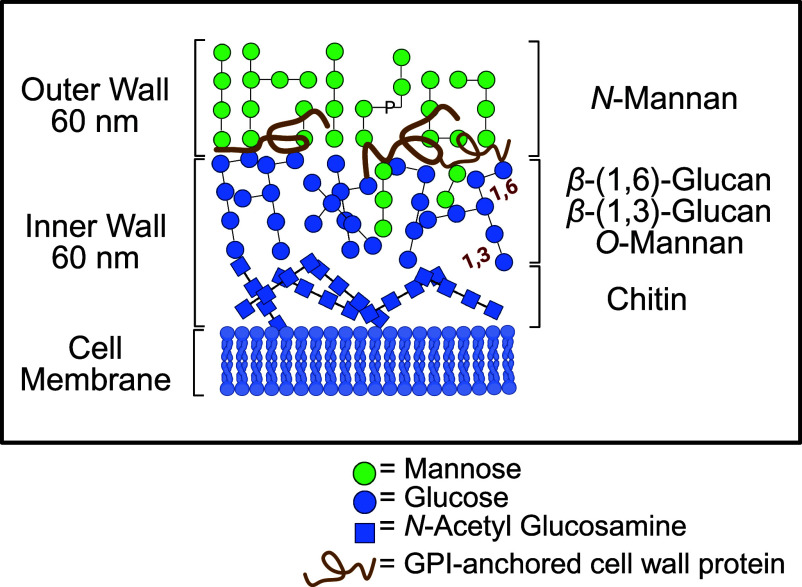
Architecture of the *Candida* cell wall. Adapted and modified from (14, 88).

The fungal cell wall consists of about 80% polysaccharides. The outer layer of the cell wall is primarily composed of highly mannosylated glycoproteins. The branched backbone chain of an *α*-(1, 6)-linked mannan (Man) is linked to proteins by *N*-glycosidic bonds via a largely conserved triantennary complex in which two *β*-(1,4)-N-acetyl-D-glucosamine (GlcNAc) units form an amide bond to asparagine residues of the polypeptide (12). The very large branched *N*-mannan structure consists of mannose residues with *α*-(1,2), *α*-(1,3), *β*-(1,2), *β*-(1,6), and *α*-(1,6)-linked mannose units and phosphodiester bonds that link both *α*- and *β*-(1,2)*-*mannose. Chitin, an unbranched homopolymer of GlcNAc, and *β*-(1,3)- and *β*-(1,6)-glucans (Glc) are the main components of the inner layer. The *β*-(1,3)-glucans form a triple-helix with the *β*-(1,6)-glucan as branches. The cell wall proteins are mostly GPI-anchored via a C-terminal ω-site to *β*-(1,6)-glucan (Fig. 1) (13–15).

The immune response to fungal pathogens involves both innate and adaptive components and is crucial for effective immunization and detection strategies (16). Invasive *Candida* infections predominantly occur in patients with compromised natural barriers to infection, e.g., in patients with central lines or after surgery (17). Fungal cell wall glycans are major pathogen-associated molecular patterns (PAMPs) that modulate host immunity. Once epithelial barriers are breached, innate immune cells—including neutrophils, monocytes, macrophages, NK cells, and dendritic cells (DCs)—recognize fungal PAMPs via pattern recognition receptors (PRRs), triggering phagocytosis and inflammation (16, 18–22). DCs also bridge to adaptive immunity by presenting fungal antigens to T cells, promoting TH1 and TH17 responses that are key to antifungal defense (20, 23).

Fungi have developed various strategies to evade immune defenses by masking PAMPs such as *β*-(1,3)-glucan and chitin, secreting effector proteins that modulate host immune responses, and producing immunosuppressive molecules like gliotoxin, which can impair or induce apoptosis in immune cells (24, 25).

While the role of humoral immunity in fungal defense has been debated, recent studies highlight the significant contribution of antibodies in restricting fungal burden and promoting clearance through mechanisms such as opsonization, toxin neutralization, inhibition of adherence and biofilm formation, complement activation, and antibody-dependent cellular cytotoxicity (ADCC) (26–33). These findings underscore the potential of glycan-based vaccine antigens in eliciting protective antibody responses against invasive candidiasis.

Glycoconjugate vaccines—polysaccharides covalently attached to a carrier protein—overcome the limitations of polysaccharide antigens by generating T cell-dependent immunity and long-term protection (34, 35). In several studies, glycoconjugates proved immunogenic and in some cases produced protective antibodies against *Candida* (36–42), but no human antifungal monoclonal antibody or vaccine has been approved to date and preventive strategies primarily involve antifungal prophylaxis. Developing a glycoconjugate or monoclonal antibody vaccine to prevent invasive candidiasis in high-risk patients remains a critical goal (43).

Methods to detect *Candida* spp. in clinical specimens differ in their specificity, sensitivity, speed, and accuracy. Culture-based methods are most commonly used diagnostic tools and are recommended by the latest global guideline for the diagnosis and management of candidiasis (44), but have limited sensitivity and long turnaround times. Serum *β*-D-glucan (BDG) testing is often recommended in combination with clinical parameters or other diagnostic tools, such as radiography, microscopy, culture, or PCR from sterile samples (44), because of relatively frequent false-positivity due to the detection of dietary glucans and cross-reactivity with certain bacteria (45, 46). The FDA-approved BDG assay, Fungitell® was considered useful for excluding invasive fungal infection (47). Both BDG assays have a long hands-on time and require special equipment. Other biomarkers for the diagnosis of candidemia and invasive candidiasis, such as serum mannan antigen assays combined with antimannan antibody assays are also limited by low sensitivity and specificity (44, 48–51) and several different binding epitopes of the monoclonal antibody EBCA-1 used in a commercial assay for the detection of *Candida* mannan were reported and contradict each other (51–54). A reliable, easy-to-perform, and prompt test to diagnose invasive candidiasis is urgently needed (55, 56). A highly specific and sensitive lateral flow test (LFT) for the detection of *Candida* would allow for a simple device without specialized and costly equipment (57).

A constant interaction between the immunocompetent host and the pathogen *C. albicans* was shown with a serological profiling of candidemia using a protein microarray (58). The use of crude preparations of *Candida* antigens makes it difficult to study the immune response for the development of reliable detection tests and vaccines. The use of pure, well-defined synthetic glycans on glycan microarrays offers a high-throughput method for rapid analysis of serum antiglycan antibodies and discovery of new biomarkers (59).

Here, we report the search for serum antibodies using glycan microarrays containing pure, well-defined *Candida*-related synthetic glycans to better understand the immune response to *Candida* infections as a basis to develop diagnosis and prevention strategies.

## Results

### Collection of Synthetic Glycans on Glycan Microarray.

**Fig. 2.**
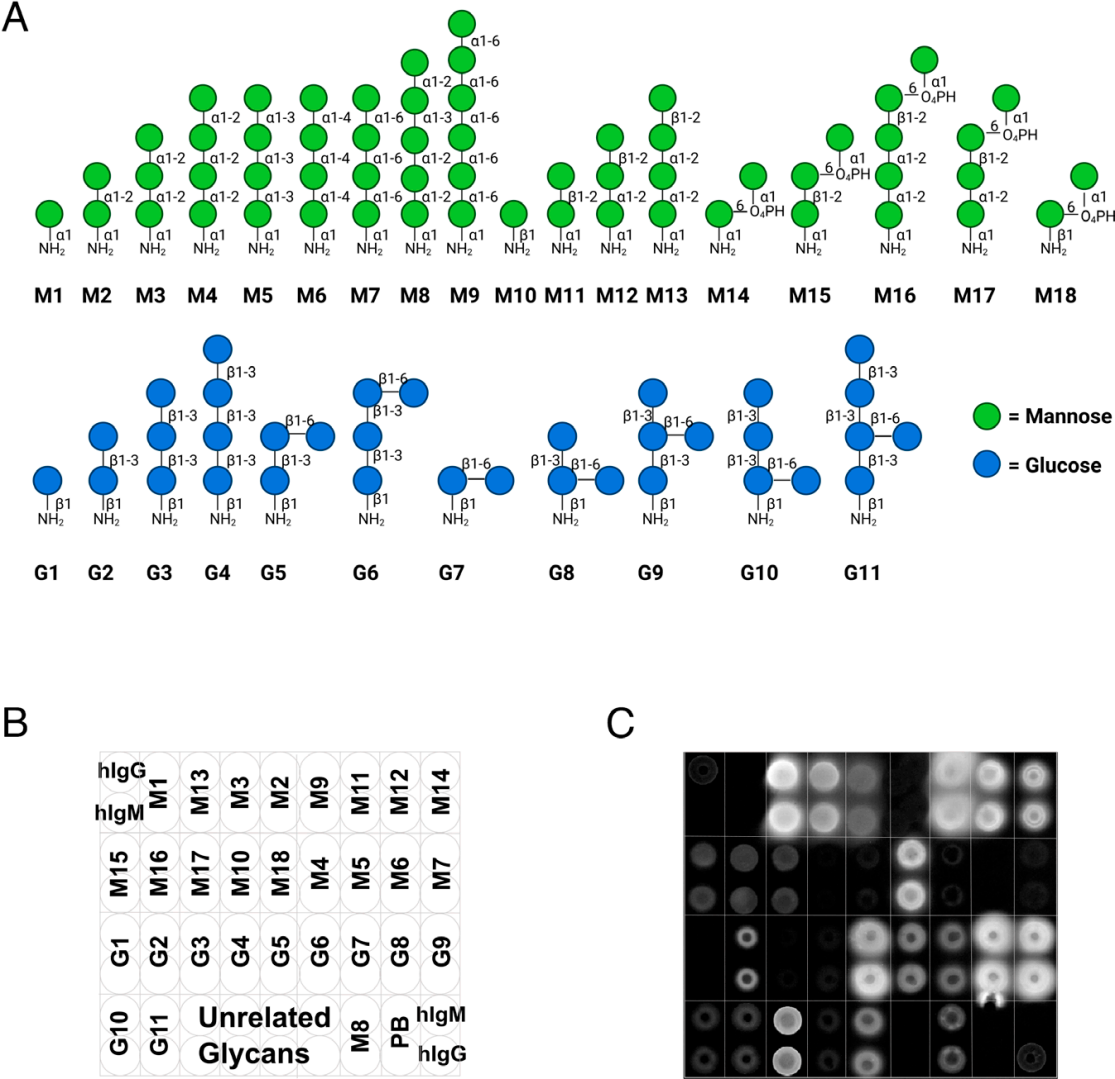
Synthetic glycans in glycan microarray analysis. Schematic representation of the synthetic glycans (*A*), glycan microarray printing pattern (*B*) and exemplary binding pattern of human serum to immobilized synthetic glycans (*C*).

We synthesized 29 well-defined *Candida*-associated mannoses and *β*-glucans with different linkages and branches (Fig. 2*A*) following established protocols (60–63). The synthetic glycans were equipped with an aminopentanol linker for immobilization on N-hydroxy-succinimide (NHS)-activated glass slides in a defined pattern (Fig. 2*B*) (64). Upon incubation with sera, the antibodies bound to the glycans were detected by incubation with a fluorophore-labeled secondary antibody (Fig. 2*C*).

### Glycan Microarray Analysis of Human Sera.

To detect antibodies against the synthetic glycans, we screened sera of humans with invasive candidiasis by different *Candida* species (n = 50) at different time points of the infection using sera of *Candida*-negative individuals as controls (n = 30). To determine the best serum dilution for our glycan microarray experiments, we tested a range of dilutions (1:10 to 1:1,000) using sera from two patients and measured IgG binding to selected glycans (**M4**, **M8**, and **M13**) (*SI Appendix*, Fig. S6*A*). Interestingly, the signal intensity increased from 1:10 up to 1:100 but then dropped at higher dilutions. This pattern is consistent with a prozone effect, where too much antibody at low dilution can interfere with optimal binding (65–67). To rule out nonspecific interactions, we tested a control isotype antibody across a wide concentration range, which showed no detectable background binding (*SI Appendix*, Fig. S6*B*). Based on these results, we chose 1:100 as the optimal dilution for the main study, balancing strong signal with minimal background.

**Fig. 3.**
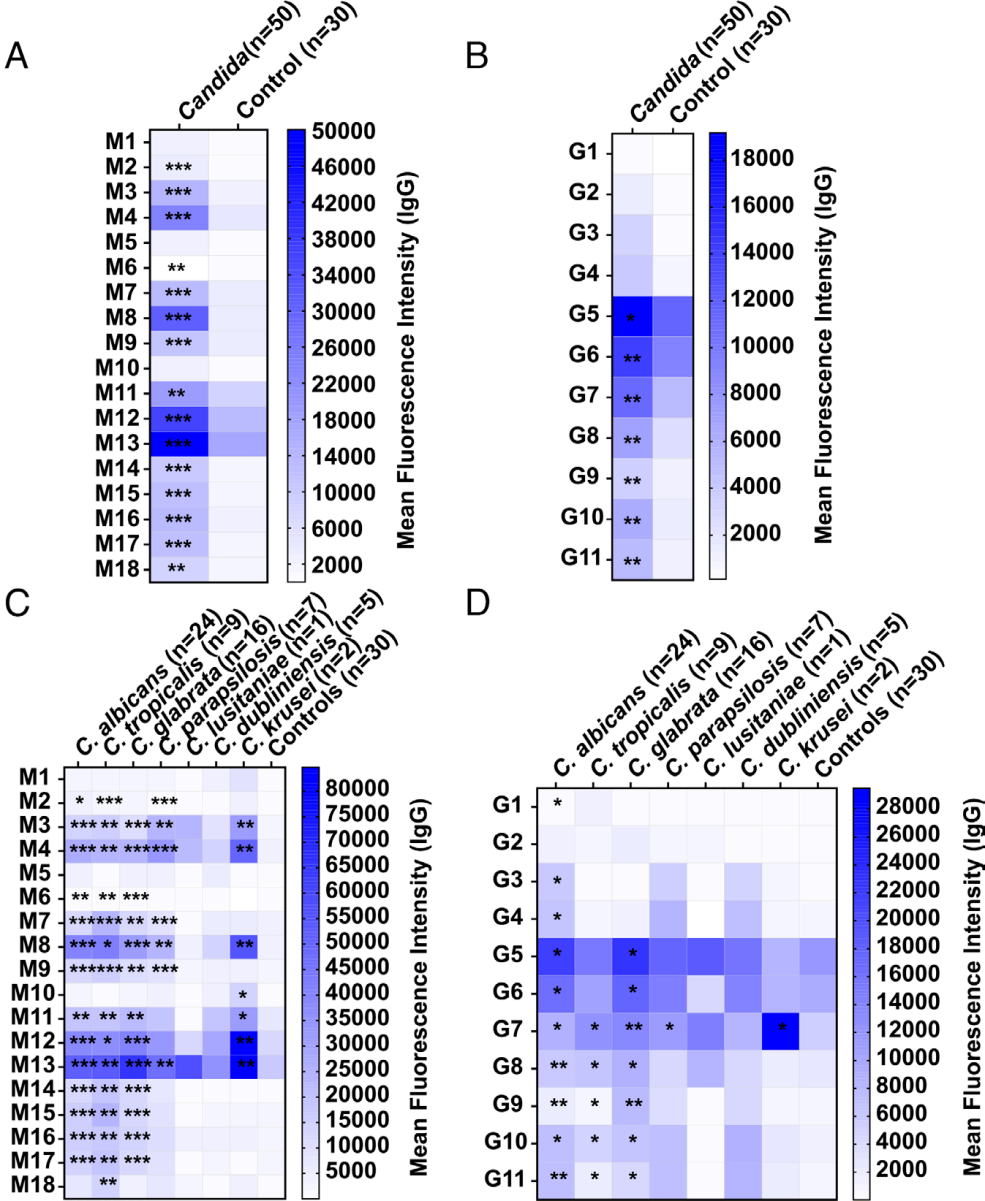
Mean fluorescence intensity of IgG antibody binding to synthetic glycans in humans. Mean fluorescence intensity of IgG antibodies in sera derived from humans with invasive candidiasis, binding to synthetic mannans (*A*) or *β*-glucans (*B*). Mean fluorescence intensity of IgG antibodies in sera derived from humans with different *Candida* spp. infections, binding to synthetic mannans (*C*) or *β*-glucans (*D*). A serum dilution of 1:100 was used. Values represent mean. Differences were tested for significance to noninfected controls (*A*–*D*) using multiple Mann–Whitney test with ***P < 0.001, **P < 0.01, and *P < 0.05.

Patients with invasive *Candida* infections showed significantly higher IgG antibody levels in their sera toward mannans **M2**, **M3**, **M4**, **M6**, **M7**, **M8**, **M9**, **M11**, **M12**, **M13**, **M14**, **M15**, **M16**, **M17**, **M18** (Fig. 3*A*) and *β*-glucans **G5**, **G6**, **G7**, **G8**, **G9**, **G10**, **G11** (Fig. 3*B*) than controls. The mean fluorescence intensities of IgG antibodies in *Candida* patients, categorized by individual species (Fig. 3 *C* and *D*), revealed that individuals infected with *C. albicans* (n = 24) exhibit additional IgG antibodies against **G1**, **G3**, and **G4**. Patients infected with *C. glabrata* (n = 16) did not show an increased IgG antibody level against **M2**. *C. parapsilosis* (n = 7) patients did not show IgG antibodies against **M6**, **M11**, **M12**, **M14**, **M15**, **M16**, **M17**, **G5**, **G6**, **G8**, **G9**, **G10**, **G11**. *C. lusitaniae* (n = 1) and *C. dubliniensis* (n = 5) patients showed no significant IgG binding toward the synthetic glycans. Humans infected with *C. krusei* (n = 2) did not show an increased IgG antibody level against **M2**, **M6**, **M7**, **M9**, **M14**, **M15**, **M16**, **M17**, **G5**, **G6**, **G8**, **G9**, **G10**, and **G11**, but in contrast to the other patients, showed IgG antibodies against **M10**. IgG antibodies against **M18** could only be detected in humans infected with *C. tropicalis* (n = 9).

**Fig. 4.**
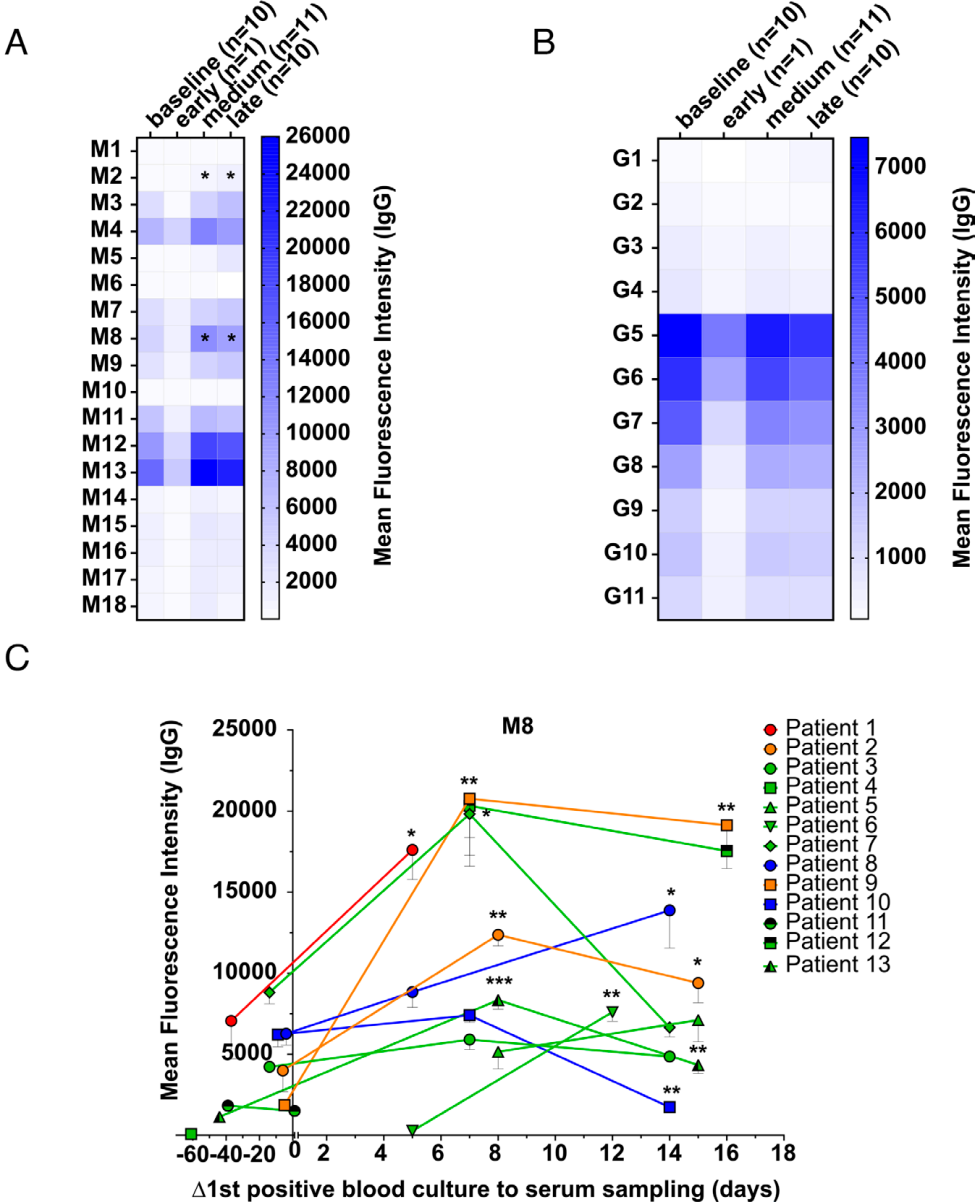
Mean fluorescence intensity of IgG antibody binding to synthetic glycans in humans monitored over time. Mean fluorescence intensity of IgG antibodies in sera derived from humans before (baseline) or at an early (1 d), medium (5 to 8 d) or late (12 to 16 d) timepoint after positive blood culture with invasive *Candida* spp., binding to synthetic mannans (*A*) or *β*-glucans (*B*) or glycan **M8** of individual patients infected with *Candida dubliniensis* (red), *Candida albicans* (orange), *Candida glabrata* (green), or *Candida parapsilosis* (blue) (*C*). Two patients with invasive *C. glabrata* infection were not screened after the first positive blood culture, and three were not screened before the first positive blood culture. A serum dilution of 1:100 was used. Values represent mean (*A* and *B*) with SEM (*C*). Differences were tested for significance to baseline using multiple Mann–Whitney test with ***P < 0.001, **P < 0.01 and *P < 0.05 (*A* and *B*) or to the first timepoint of sampling of the individual patients using Welch’s *t* test with ***P < 0.001, **P < 0.01 and *P < 0.05 (*C*).

The IgG antibody levels of *Candida* patients were also monitored over time (Fig. 4). Patients showed a significant increase in IgG antibodies against **M2** and **M8** five to eight days after the first positive blood culture (Fig. 4*A*). IgG antibodies against *β*-glucans were detectable in patients before the first positive blood culture and the level did not change over time (Fig. 4*B*). Seven out of thirteen *Candida* patients (one out of one *C. dubliniensis*, two out of two *C. albicans*, three out of eight *C. glabrata,* and one out of two *C. parapsilosis* infected patients) showed increased levels of IgG antibodies against **M8** compared to the first time point of serum sampling of the respective patient (Fig. 4*C*). Patients with invasive *Candida* infection showed lower IgG antibody levels against *β*-glucans compared to mannan structures (Figs. 3 and 4).

### Glycan Microarray Analysis of Mice Sera.

To investigate the antiglycan antibody level over time, it is necessary to eliminate any potential confounding factors, including those associated with other diseases. In addition, patients suffering from candidemia are often immunocompromised. Therefore, the study of in vivo infection of immunocompetent hosts helps to identify the key epitopes for an effective antifungal response.

**Fig. 5.**
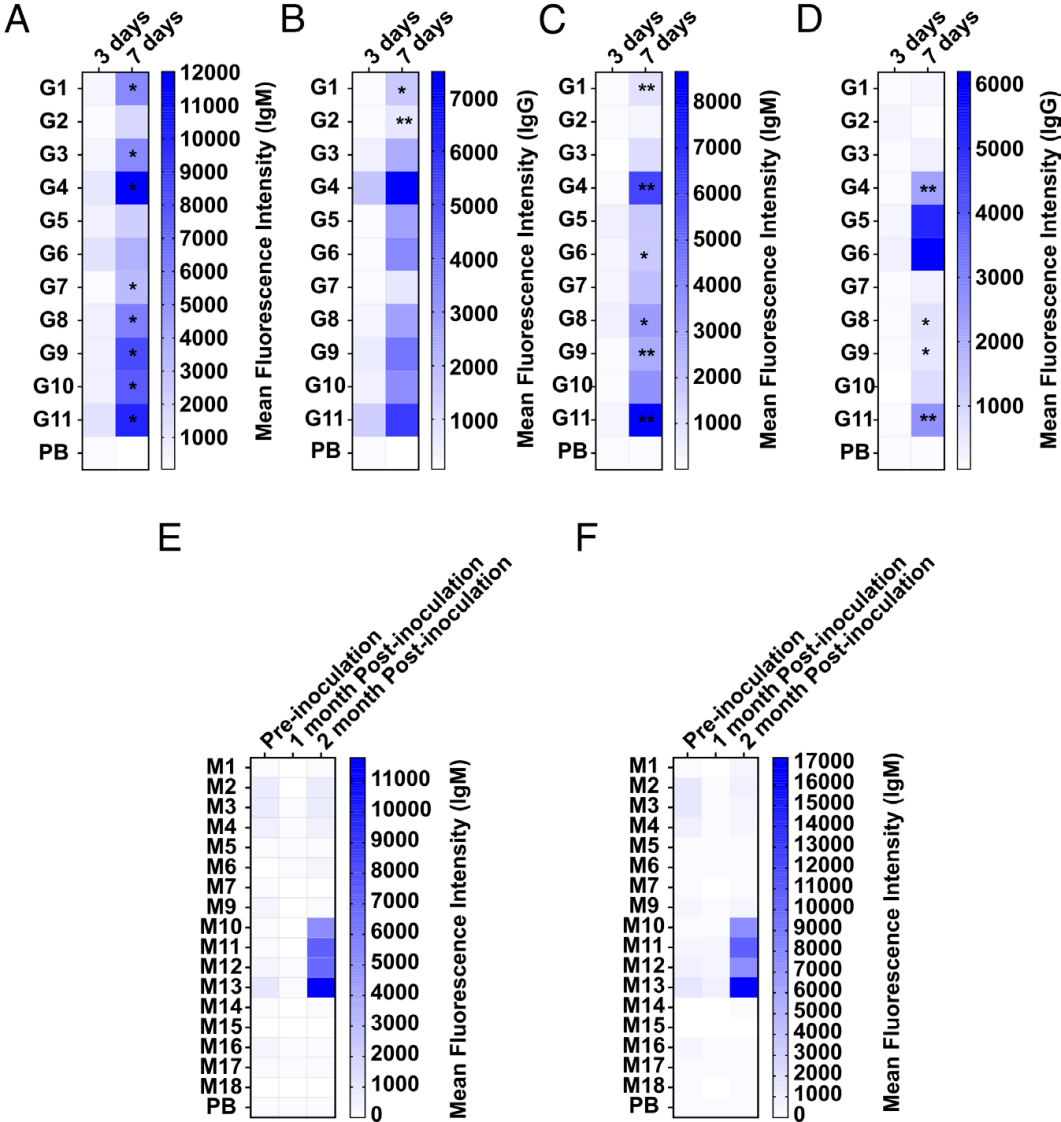
Mean fluorescence intensity of IgM and IgG antibody binding to synthetic glycans in mouse models of invasive candidiasis. Mean fluorescence intensity of IgM antibodies (*A*) and IgG antibodies (*B*) binding to synthetic *β*-glucans after three days or seven days of infection with live *C. albicans* (CWZ 10061110), and IgM antibodies (*C*) and IgG antibodies (*D*) binding after three days or seven days of infection with live *C. auris* (CWZ 10051896) (belonging to clade I). Mean fluorescence intensity of IgM antibodies binding to synthetic mannans after one month or two months of inoculation with killed *C. auris* NCPF13001#16 (clade 1) (*E*) or *C. auris* VPCI479/P/13 (clade 1) (*F*). A serum dilution of 1:100 was used. Values represent mean. Differences were tested for significance to three days of infection (*A*–*D*) or preinoculation (*E* and *F*) using multiple Mann–Whitney test with ***P < 0.001, **P < 0.01 and *P < 0.05.

We infected intravenously immunocompetent mice with live *C. albicans* (CWZ 10061110) and live *C. auris* (CWZ 10051896) (belonging to clade I) and killed the mice after three and after seven days. After seven days of infection with *C. albicans* IgM antibodies against **G1**, **G3**, **G4**, **G7**, **G8**, **G9**, **G10**, and **G11** (Fig. 5*A*) and IgG antibodies against **G1** and **G2** were detected (Fig. 5*B*). Mice infected with live *C. auris* showed after seven days IgM antibodies against **G1**, **G4**, **G6**, **G8**, **G9**, and **G11** (Fig. 5*C*) and IgG antibodies against **G4**, **G8**, **G9**, and **G11** (Fig. 5*D*). After three days, mice infected with *C. albicans* showed IgG antibodies against **M9** and after seven days IgM antibodies directed against **M10** and IgG antibodies binding to **M12** (*SI Appendix*, Fig. S4 *C* and *D*) were visible. Mice infected with *C. auris* showed an increase of IgM antibodies against the mannose structures **M10**, **M11**, **M12**, and **M13** after three days of infection but not after seven days, which was already reported (61, 68). After seven days, IgM antibodies against **M1** and IgG antibodies against **M9**, **M12**, and **M15** were detected (*SI Appendix*, Fig. S4 *G* and *H*). None of these increases of IgM and IgG antibody levels were significant (*SI Appendix*, Fig. S4).

To determine long-term epitopes, two different *C. auris* isolates [*C. auris* NCPF13001#16 (clade 1—South Asia) and *C. auris* VPCI479/P/13 (clade 1—South Asia)] (69) were used to intraperitoneally inoculate immunocompetent mice and monitor antibody response for up to two months. Due to the long-time span of experiment, killed *Candida* was used for ethical reasons. IgM antibodies against **M10**, **M11**, **M12**, and **M13** were detected after two months of inoculation with *C. auris* isolates *C. auris* NCPF13001#16 and *C. auris* VPCI479/P/13 (Fig. 5 *E* and *F*). No IgM antibodies against the *β*-glucan structures and no IgG antibodies against either mannose or *β*-glucan structures were detected after two months postinoculation (*SI Appendix*, Fig. S5).

## Discussion

The cell wall of *Candida* contains predominantly mannans and *β*-glucans (13–15). To investigate the immune response, particularly antibody production, against *Candida* infections as a foundation for developing diagnostic and preventive strategies based on synthetic glycans or glycan-associated antibodies, we screened sera from infected and noninfected humans and mice using glycan microarray. By employing well-defined synthetic glycans, we were able to identify glycans that represent important antigenic targets for IgM and IgG antibodies produced in response to *Candida* infections. An unexpected yet well-characterized feature of our titration data was the nonlinear binding pattern observed at low serum dilutions (*SI Appendix*, Fig. S6*A*). Specifically, fluorescence signals increased from 1:10 to 1:100 but decreased at both higher and lower dilutions. The reduced signal at the lowest dilution (1:10) is likely due to a prozone effect (also known as the high-dose hook effect), which occurs when antibody concentrations are so high that they interfere with effective antigen binding or proper complex formation (65–67). This effect has been widely described in immunoassays, including ELISAs and lateral flow formats and can lead to underestimation of true binding levels if not accounted for. Our control experiments with an isotype antibody showed no background binding across the concentration range tested (*SI Appendix*, Fig. S6*B*), ruling out nonspecific signal contributions and further supporting the conclusion that the reduced signal at low dilution reflects biological interference rather than technical artifact. These results validate the choice of 1:100 dilution for the main screen and highlight the importance of titer optimization when interpreting microarray data. As described previously (58, 70, 71), we found both anti-mannan and anti-*β*-glucan antibodies in sera from noninfected humans (Figs. 3 and 4). *Candida* species colonize the skin as well as the mucous membranes of the gastrointestinal tract, the urogenital tract, and the respiratory tract, and thereby stimulate the immune system, like the commensal *C. albicans* in the intestine, which leads to systemic expansion of fungal-specific Th17 CD4^+^ T cells and cross-protection against other fungal pathogens (72–74). Recent work has shown that healthy individuals often carry circulating IgG against fungal cell wall components, likely due to mucosal colonization and antigen translocation (75), consistent with our findings. Natural antibody responses, such as germinal center–independent IgM, may also play a role in baseline antifungal reactivity (76). Therefore, we compared the sera of patients with invasive *Candida* infection with the noninfected controls to define infection-specific markers.

Not all patients with invasive *Candida* spp. infection showed increased antibody levels toward the glycans (Fig. 4*C* and *SI Appendix*, Fig. S3), presumably because of delayed, reduced, or absent immunological responses in severely immunocompromised patients (71, 72). Accordingly, a comparison was conducted between the mean values of the infected group and the control group, with antibody levels monitored over time before and after positive blood culture.

Previous studies have shown that the *β*-glucan content in the fungal cell wall is higher than that of mannan, yet it elicits a weaker immune response (71). One possible explanation for this observation is the shielding effect of *N*-mannans, which conceals the majority of *β*-glucan from immune recognition (77–80). This is also underscored by our analyses, where a lower antibody level was found in patients with invasive *Candida* infection against *β*-glucans compared to mannan structures (Figs. 3 and 4). The patients were infected with *Candida* despite a high antibody level against *β*-glucans (Fig. 4*B*), which suggests that the antibodies are not protective. *β*-Glucan shielding by *N*-mannans could also explain this. However, it has been shown that passive protection against *Candida* infection can be mediated by antibodies reacting to *β*-(1,3)- and *β*-(1,6)-glucan (81). Mannans are considered the most important antigen for the humoral immune response, as it is a major component of the cell wall and is one of the main *Candida* antigens circulating during infection (48).

Immunocompetent mice infected with live *C. albicans* or *C. auris* first produced IgM antibodies against *β*-glucan structures and later on IgG antibodies against *β*-glucans. This leads to the assumption that the immune response toward *β*-glucan structures is the initial antibody defense mechanism (Fig. 5). An infection with *C. albicans* leads to an overall higher IgG antibody level toward *β*-glucans compared to an infection with *C. auris*. This is consistent with previously reported findings that *C. auris* has a distinct cell wall composition in which an outer mannan layer shields the inner *β*-(1,3)-glucan (79). *C. auris* also act differently than *C. albicans* and elicit less strong innate immune responses (82). After three days of infection with *C. albicans*, mice developed IgG antibodies against the mannose structure **M9**. By day seven, additional responses were detected, including IgM antibodies against **M10** and IgG antibodies binding to **M12** (*SI Appendix*, Fig. S4 *C* and *D*). In contrast, mice infected with *C. auris* exhibited an early IgM response after three days against **M10**, **M11**, **M12**, and **M13**. This early IgM reactivity diminished by day seven, consistent with previous findings (61, 68). By day seven, these mice instead showed IgM reactivity against **M1** and IgG responses to **M9**, **M12**, and **M15** (*SI Appendix*, Fig. S4 *G* and *H*). However, none of these observed increases in IgM or IgG antibody levels reached statistical significance (*SI Appendix*, Fig. S4), suggesting that the humoral response to these mannose structures during early infection is limited or variable.

In a longer term model utilizing killed *C. auris* we detected IgM antibodies directed against oligo-mannoses containing a terminal *β*-(1,2)-linkage (**M10**, **M11**, **M12**, **M13**) in exposed mice, which was already reported (61, 68), suggesting a role in the activation of antifungal immune response (Fig. 5 *E* and *F*), although the rise in antibody level was moderate. Two months after inoculation with killed *Candida*, no IgG antibodies against mannose structures were detected (*SI Appendix*, Fig. S5), suggesting that the formation of IgG antibodies takes longer due to a lower dose or killing. The antibody response after inoculation with *C. auris* VPCI479/P/13 leads to a higher IgM antibody level toward mannans than an infection with *C. auris* NCPF13001#16, supporting the results that the mannose composition differs also inside a clade (83).

*β*-glucan structures tend to be homopolymers composed of *β*-(1,3)- and *β*-(1,6)- or *α-*(1,3)-glucan, therefore, less complex than mannans, which are heteropolymers that consist of many different mannose linkages (14, 15, 84). This simpler structure can explain why antibodies toward glucans are produced first (Fig. 5 and *SI Appendix*, Fig. S4) and are present in the human control samples (Figs. 3 and 4).

Only donors infected with *C. krusei* showed antibodies against the *β*-(1,3)-mannose monomer (**M10**), while no antibodies were detected in those infected with other *Candida* species (Fig. 3). The binding to the mannose monomer (**M10**) of antibodies in sera from patients infected with *C. krusei* may suggest conformational effects related to glycan presentation on the array. The presentation via a linker and solid-phase attachment may allow for spatial stabilization or clustering that mimics part of a native epitope. Antibody production toward **M10** mannose monomer is unique to *C. krusei* and, therefore, potentially a good biomarker. On the other hand, all the infected humans showed significantly higher antibody levels against the pentasaccharide antigen *α*-(1,2)Man-*α*-(1,3)Man-*α*-(1,2)Man-*α*-(1,2)Man-*α*-(1,2)Man (**M8**) and the tetrasaccharide antigen *β*-(1,2)Man-*α*-(1,2)Man-*α*-(1,2)Man-*α*-(1,2)Man (**M13**) compared with the noninfected controls, suggesting a diagnostic option (Fig. 3). The patients monitored over time showed an increased antibody level against **M8** and not against *α*-(1,2)Man-*α*-(1,2)Man-*α*-(1,2)Man-*α*-(1,2)Man (**M4**) (Fig. 4*A*), leading to the assumption that the combination of the *α*-(1,2) and *α*-(1,3) linkages of the mannose-structures is important for the immune recognition. The small sample size for patients infected with *C. lusitaniae* and *C. krusei* limits the significance of these subgroups. Future studies with larger cohorts are needed to confirm these observations.

Humans infected with *C. albicans*, *C. tropicalis,* and *C. glabrata* showed, in contrast to *C. parapsilosis*, *C. lusitaniae*, *C. dubliniensis,* and *C. krusei* species, IgG antibodies against the phosphodiester-linked mannose-structures **M14**, **M15**, **M16**, **M17**, and **M18** (Fig. 3). A possible explanation for this observation is due to the absence of these structures in these species or due to the shielding of the phosphodiester linker in the cell wall.

Infected humans, as well as infected mice, showed a significant increase in antibodies against the tetrasaccharide antigen *β*-(1,2)Man-*α*-(1,2)Man-*α*-(1,2)Man-*α*-(1,2)Man (**M13**) and the pentasaccharide antigens *α*-(1,2)Man-*α*-(1,3)Man-*α*-(1,2)Man-*α*-(1,2)Man-*α*-(1,2) Man (**M8**) and *β*-(1,3)Glc-*β*-(1,3)Glc-*β*-(1,3)[Glc-*β*-(1,6)]Glc-*β*-(1,3)Glc (**G11**). The delayed antibody production argues against the use of synthetic glycans for the early detection of *Candida*. Because of the presence of *β*-glucan antibodies in the control groups, the *β*-glucan shielding (77–79), and previous studies, which showed that serum *β*-Glc (BDG) assays are not sensitive enough (45, 85), mannan structures are more attractive antigen candidates for the detection, e.g., through a monoclonal antibody LFT, and development of glycoconjugate or monoclonal antibody vaccines against all the *Candida* species tested.

Species-specific differences in *Candida* cell wall glycans, particularly mannans and glucans, are important for host immunity and should be explored further to improve diagnostics and vaccine design. While we focused on identifying glycan targets that could serve as potential biomarkers or vaccine candidates by analyzing total IgG responses, analyzing IgG subclasses could add valuable insight and is an important direction for future studies.

## Materials and Methods

### Synthetic *Candida*-Related Glycans.

The mannans and *β*-glucans were synthesized following established protocols (60–63).

### Isolation of Human Serum From Whole Blood.

Serum collection tubes (Sarstedt, Germany) were centrifuged at 2,000×*g* for 15 min at room temperature to separate serum from clotted blood. Serum was aliquoted, frozen, and stored at −80 °C for future use.

### Study Participant Details.

Serum samples were collected from patients diagnosed with proven candidemia or other forms of invasive candidiasis, as defined by the 2020 European Organization for Research and Treatment of Cancer/Mycoses Study Group (EORTC/MSG) consensus criteria (86) (Table 1). The study protocol (Ethics ID: 08-160) was approved by the Institutional Review Board (IRB) of the University of Cologne, Germany. All participants provided written informed consent before enrollment.

**Table 1.** Demographics of candidemia patients of the University of Cologne cohort

Total patients	47
Age, years (mean, range)	60.6 (21–86)
Sex (M/F)	30M, 17F
Time from 1^st^ positive blood culture to serum sampling, days (median, range)	78.1 (3–692)
Mixed infection (≥2 *Candida* species)	9
Organ manifestation	
Bloodstream infection	29
Intraabdominal	13
Bone	5
Deep soft tissue	2
Chronic disseminated	2
Urinary tract	5
Outcome
Death	15
Survival	32
Unknown	1

Serum samples of 13 patients with invasive *Candida* spp. infection were drawn as part of a prospective observational cohort study conducted at Charité—Universitätsmedizin Berlin, Germany (87) (Table 2). Samples were collected before, 7 ± 2 d or 14 ± 2 d after first *Candida* spp. positive blood culture. The study was approved by the Institutional Review Board of Charité (EA2/066/20) and conducted in accordance with the Declaration of Helsinki and guidelines of Good Clinical Practice (ICH 1996). Written informed consent was either obtained before sampling or retrospectively after recovery, if patients were mechanically ventilated at the time of sampling.

**Table 2.** Demographics of invasive *Candida* spp. patients of Charité cohort

Total patients	13
Total samples	31
…baseline	10
…day 7 ± 2	11
…day 14±2	10
Sex (M/F)	12M, 1F
Age, years (mean, range)	61, 27–78
Time from hospital admission to 1^st^ positive blood culture, days (median, range)	20, 7–72

*Candida*-negative controls were recruited as part of two prospective observational studies conducted at Charité—Universitätsmedizin Berlin (EA2/066/20 and EA4/245/20) (Table 3). Written informed consent was either obtained before sampling or retrospectively after recovery, if patients were mechanically ventilated at the time of sampling. Although detailed clinical histories or microbiologic records were not available to definitively rule out previous *Candida* exposure, all control individuals were not infected at the time of sampling and had no known history of invasive fungal infections.

**Table 3.** Demographics of *Candida*-negative controls of the Charité cohort

Total patients	15
Total samples	20
Sex (M/F)	11 M, 4F
Age, years (mean, range)	57.2, 22–78

### Mice Infection With Inactivated *C. auris* NCPF13001#16 or VPCI479/P/13.

Animal experiments performed at the University of Exeter conformed to the local ethical review committee and were performed in compliance with animal research ethical regulations under the UK Home Office Project licence number P6A6F95B5.

Male C57BL/6 mice (8 to 10 wk old; Charles River) were housed in ventilated cages (4 per cage) and supplied with food and water ad libitum. Mice were maintained on a 12 to 12 h dark-light cycle at 20 to 24 °C and relative humidity of 55 ± 15%. On days 0, 14, 30, 44, and 56, mice were administered intraperitoneal (i.p.) injections of 200 µL containing 10^7^ cells/mL of *C. auris* clinical isolates NCPF13001#16 or VPCI479/P/13. These *C. auris* cell suspensions were prepared by harvesting exponentially growing cells in YPD, fixing them in 50 mM thimerosal, and washing and resuspending them in phosphate-buffered saline. After inoculation with the fixed *C. auris* cells, the mice were monitored daily for the development of clinical symptoms. Peripheral blood was collected by tail nicking to measure antibody levels during the time course. Mice were culled on day 60 via cardiac puncture under anesthetic to allow for the collection of blood. Serum was isolated from each blood samples by centrifugation (7,000 rpm for 5 mins) and stored in a freezer (−80 °C).

### Mice Infection with live *C. albicans* and live *C. auris*.

The study used male C57Bl6 mice (7 to 8 wk old) from the Pasteur Institute (Athens, Greece, EL 25 BIObr 011). Mice acclimated for seven days, housed at 21 °C with a 12-h light/dark cycle, and provided ad libitum food and water. Paracetamol was used for analgesia to avoid immune interactions. Healthy mice were i.v. challenged via the tail vein with 1x10^7^ CFU/mouse of *C. albicans* CWZ 10061110 (n = 11) or *C. auris* CWZ 10051896 (n = 10) under light methoxyflurane anesthesia. Mice were randomized, and survival monitored for 14 d. At days 3 and 7 postinoculation, mice (n = 4 to 5 per group/timepoint) were killed using intramuscular ketamine. Serum was isolated from each blood sample by centrifugation (7,000 rpm for 5 mins) and stored in a freezer (−80 °C).

### Detection of Anti-*Candida*-Glycan Antibodies Using Microarrays.

The glycans were dissolved at 0.1 mM in 50 mM sodium phosphate buffer pH 8.5 and printed in 64 identical fields to *N*-hydroxysuccinimide (NHS) ester-activated hydrogel glass slides (TRIDIA™ Activated Slides, Surmodics) using a noncontact sciFLEXARRAYER S12 microarray spotter (Scienion, Berlin, Germany). After incubation overnight in a humidified box, the remaining NHS groups of the slides were quenched with ethanolamine. The slides were blocked with 1% (w/v) bovine serum albumin (BSA) in phosphate-buffered saline (PBS) and a 64-well incubation gasket (FlexWell Grid, Grace Bio Labs) was attached. The slides were incubated with human or mice serum diluted 1:100 in 1% BSA-PBS for 1 h at 37 °C. To determine the optimal dilution, 1:10, 1:50, 1:100, 1:500, and 1:1,000 dilutions of sera of two patients were tested. A control isotype antibody (goat antihuman IgG Fc-AF647 (SouthernBiotech, Cat. 2048-31)) was also tested at several concentrations (1.25, 50 and 100 µg/mL) to evaluate nonspecific binding. After three washes with PBS containing 0.1% (v/v) Tween-20 (PBS-T), the slides were incubated with goat antihuman IgG Fc-AF647 (SouthernBiotech, Cat. 2048-31), goat antihuman IgM (µ) FITC conjugate (Invitrogen, Cat. H15001), Alexa Fluor® 488 AffiniPure™ rabbit antimouse IgG, Fcγ fragment specific (Jackson Immuno Research, Cat. 315-545-008), Alexa Fluor™ 647 goat antimouse IgM (µ chain) (Invitrogen, Cat. A21238) diluted 1:400 for 1 h at 37 °C. The slides were washed twice with PBS-T. After removing the gasket, the slides were washed once with PBS and once with water. The dried slides were scanned with an InnoScan 1100 Fluorescence Scanner (Innopsys). Intensities were evaluated with Mapix 9.1.0 (Innopsys).

### Data Analysis.

Results were analyzed using GraphPad Prism 10.4.0 (GraphPad Software, Inc.). Statistical significance was determined as described in the figure legends. *P* < 0.05 was considered statistically significant. All heat maps depict mean, and all graphs depict mean ± SEM.

## Supplementary Material

Appendix 01 (PDF)

## Data Availability

All study data are included in the article and/or *SI Appendix*.
