# Supplementary material for: Glycan microarray analysis of Candida-related antibodies in human and mice sera guides biomarker discovery and vaccine development
Source: Proc Natl Acad Sci U S A. 2025 Sep 25;122(39):e2505340122. doi: 10.1073/pnas.2505340122 (PMC12501125; doi:10.1073/pnas.2505340122)
Supplement: Supplementary file 1 — Appendix 01 (PDF) [file pnas.2505340122.sapp.pdf]

PNAS

Supporting Information for

Glycan Microarray Analysis of *Candida* Related Antibodies in Human and Mice Sera Guides Biomarker Discovery and Vaccine Development

Emelie E. Reuber,[1,2] Emer Hickey,[3] Arnab Pradhan,[3] Rosanne Sprute,[4,5,6] Tilman Lingscheid,[7] Pinkus Tober-Lau,[7] Ian Leaves,[3] Mark H. T. Stappers,[3] Florian Kurth,[7,8,9] Mariolina Bruno,[10] Mihai G. Netea,[10,11] Leif E. Sander,[7,9,12] Oliver Cornely,[4,5,6,13] Neil A. R. Gow,[3] Alistair J. P. Brown,[3] Rajat K. Singh,[1,2] Sabrina Omoregbee-Leichnitz,[1,2] Eric T. Sletten,[2] José Danglad-Flores,[2] Peter H. Seeberger[1,2*]

[1] Institute of Chemistry and Biochemistry, Freie Universität Berlin; Berlin, Germany.

[2] Max Planck Institute of Colloids and Interfaces; Potsdam, Germany.

[3] Medical Research Council Centre for Medical Mycology at the University of Exeter, University of Exeter, Geoffrey Pope Building, Stocker Road, Exeter, EX4 4QD, UK.

[4] University of Cologne, Faculty of Medicine and University Hospital Cologne, Institute of Translational Research, Cologne Excellence Cluster on Cellular Stress Responses in Aging-Associated Diseases (CECAD), Cologne, Germany.

[5] University of Cologne, Faculty of Medicine and University Hospital Cologne, Department I of Internal Medicine, Center for Integrated Oncology Aachen Bonn Cologne Duesseldorf (CIO ABCD) and Excellence Center for Medical Mycology (ECMM), Cologne, Germany.

[6] German Centre for Infection Research (DZIF), Partner Site Bonn-Cologne, Cologne, Germany.

[7] Department of Infectious Diseases and Critical Care Medicine, Campus Virchow-Klinikum and Campus Charité Mitte, Charité - Universitätsmedizin Berlin, Corporate Member of Freie Universität and Humboldt-Universität zu Berlin; Berlin, Germany.

[8] Centre de Recherches Médicales de Lambaréné CERMEL, 242 Lambaréné, Gabon.

[9] German Center for Lung Research (DZL), Berlin, Germany.

[10] Department of Internal Medicine and Radboud Center for Infectious Diseases, Radboud University Medical Center; Nijmegen, the Netherlands.

[11] Department of Immunology and Metabolism, Life and Medical Sciences Institute, University of Bonn, Bonn, Germany.

[12] Berlin Institute of Health at Charité - Universitätsmedizin Berlin; Berlin, Germany.

[13] University of Cologne, Faculty of Medicine and University Hospital Cologne, Clinical Trials Centre Cologne (ZKS Köln), Cologne, Germany.

*Corresponding author: Peter H. Seeberger

Email: Peter.Seeberger@mpikg.mpg.de

This PDF file includes:

Supporting text
Figures S1 to S6
SI References

Supporting Information Text

1. General materials and methods

All chemicals used were reagent grade and used as supplied unless otherwise noted. The automated syntheses were performed on a home-built synthesizer developed at the Max Planck Institute of Colloids and Interfaces. Analysis and purification by normal and reverse phase HPLC were performed using the Agilent 1260 series equipped with a multiple wavelength detector (MWD) and an evaporative light scattering detector (ELSD). Products were lyophilized using a Christ Alpha 2-4 LD plus freeze dryer. ^{1}H, ^{13}C, COSY, and HSQC NMR spectra were recorded on a Bruker 400-MR (400 MHz) or a Varian 600-MR (600 MHz). Spectra were recorded in CDCl3 by using the solvent residual peak chemical shift as the internal standard (CDCl3: 7.26 ppm ^{1}H, 77.0 ppm ^{13}C) or in D_2O using the solvent as the internal standard in ^{1}H NMR (D2O: 4.79 ppm ^{1}H). The ^{1}H NMR was acquired without heteroatom decoupling. High-resolution mass spectra were obtained using a 6210 ESI-TOF mass spectrometer (Agilent).

2. Automated Glycan Assembly

a. General materials and methods.

The automated syntheses were performed on the Peltier-based AGA synthesizer (1). All solvents used were HPLC-grade. The solvents used for the building block, activator, TMSOTf, and capping solutions were taken from an anhydrous solvent system (J.C. Meyer), and water was further removed with molecular sieves (4 Å) for moisture-sensitive solutions. Oven-dried, argon-flushed flasks were used to prepare all moisture-sensitive solutions. Activator, capping, deprotection, acidic wash, and building block solutions were freshly prepared and kept under argon during the automation run. All yields of products obtained by AGA were calculated based on resin loading. Resin loading was determined following previously established procedures (2).

b. Preparation of stock solutions.

- **Building block solution**: 6.5 equiv/glycosylation of building block were dissolved in CH_2Cl_2 (1 mL per glycosylation).
- **NIS/TfOH activator solution**: 1.35 g (6.0 mmol) of recrystallized NIS was dissolved in 40 mL of a 2:1 v/v mixture of anhydrous CH_2Cl_2 and anhydrous dioxane. Then, triflic acid (55 µL, 0.6 mmol) was added. The solution was kept at 10 °C for the duration of the automation run.
- **Fmoc deprotection solution**: A solution of 20% piperidine in DMF (v/v) was prepared.
- **Lev deprotection solution**: Hydrazine acetate (550 mg, 5.97 mmol) was dissolved in pyridine/AcOH/H_2O (40 mL, v/v, 32:8:2) and sonicated for 10 min.

- **TMSOTf solution**: TMSOTf (0.45 mL, 2.49 mmol) was added to CH_2Cl_2 (40 mL) or for glycosyl phosphate activation; TMSOTf (0.9 mL, 5.0 mmol) was added to CH_2Cl_2 (40 mL).
- **Capping solution**: A solution of 10% acetic anhydride and 2% methanesulfonic acid in CH_2Cl_2 (v/v) was prepared.

c. Modules for automated synthesis.

Module A: Resin preparation for synthesis (20 min)

All automated syntheses were performed on a 0.015 mmol scale. Based on loading, an appropriate amount of resin was placed in the reaction vessel washed with DMF and CH_2Cl_2 (3 x 3 mL for 10 s each) and swollen in 2 mL of CH_2Cl_2 for 20 min before the synthesis at the temperature selected for the first coupling. All reagent lines needed for the synthesis were washed and primed during this time.

Module B: Acidic wash with TMSOTf solution (15 min)

The reactor is drained, and the temperature is adjusted to T_1 (starting glycosylation temperature typically from -40 to -20 °C). The resin is washed with CH_2Cl_2 (5 x 3 mL for 30 s each). Upon reaching the set temperature T_1, 2 mL of CH_2Cl_2 is delivered to dilute the TMSOTf solution (1 mL) and added dropwise to the reaction vessel. After bubbling for 3 min, the acidic solution was drained, and the resin was washed with 3 mL CH_2Cl_2 for 30 s.

Action	Cycles	Solution	Amount	T (°C)	time
**Cooling**	-	-	-	-20/-40*	<10 min**
**Wash**	5	CH_2Cl_2	3 mL	-20/-40*	30 s
**Delivery**	1	CH_2Cl_2	2 mL	-20/-40*	15 s
**Delivery**	1	TMSOTf solution	1 mL	-20/-40*	3 min
**Wash**	1	CH_2Cl_2	2 mL	-20/-40*	30 s

*The temperature depends on the BB utilized.

**Time required to reach the desired temperature.

Module C: Thioglycoside glycosylation (30 - 60 min)

The building block and activator solution delivery lines to the reaction vessel are primed. The building block solution (6.5 equiv of BB in 1 mL of CH_2Cl_2 per glycosylation) was delivered to the reaction vessel. After the set temperature (T_1) was reached. The reaction was started by slowly adding the NIS/TfOH activator solution (1.0 mL). The reaction is performed in two thermal steps. Starting at T_1, the reagents incubate for a time t_1, then the temperature is linearly increased by a rate of 4 °C/min to a T_2, incubating for a t_2 (times and temperature are building block dependent). The solution was drained, and the resin was washed with CH_2Cl_2:dioxane (2:1, 3 mL for 6 s). If

required, the operation was repeated (= double coupling). The resin is finally washed with CH_2Cl_2 (2 x 3 mL for 15 s).

Action	Cycles	Solution	Amount	T (°C)	Incubation time
**Cooling**	-	-	-	T_1	-
**Delivery**	1	BB solution	1 mL	T_1	3 min
**Delivery**	1	NIS/TfOH activator solution	1 mL	T_1	3 min
**Reaction time[a]**	1			T_1 to T_2	t_1 t_2
**Wash**	1	CH_2Cl_2: dioxane (2:1)	3 mL	T_2	6 s
**Wash**	2	CH_2Cl_2	3 mL	T_2	15 s

[a] There is an offset time of 5 min between T_1 and T_2 for temperature adjustment.

Module D: Capping (30 min)

The temperature of the reaction vessel was adjusted to 30 °C while the resin was washed with DMF (3 x 3 mL for 10 s). Pyridine solution (10% in DMF) was delivered (2 mL) into the reaction vessel. After 1 min, the reaction solution was drained, and the resin was washed with CH_2Cl_2 (3 x 3 mL for 10 s). Capping solution (2 mL) was delivered into the reaction vessel. After 10 min, the reaction solution was drained, and the capping reaction was repeated once more. Finally, the resin was washed with CH_2Cl_2 (3 x 3 mL for 25 s) and once with DMF for 1 min.

Action	Cycles	Solution	Amount	T (°C)	Incubation time
**Heating**	-	-	-	30	(5 min) *
**Wash**	3	DMF	3 mL	30	10 s
**Delivery**	1	10% pyridine in DMF	2 mL	30	1 min
**Wash**	3	CH_2Cl_2	3 mL	30	10 s
**Delivery**	2	capping solution	2 mL	30	10 min
**Wash**	3	CH_2Cl_2	3 mL	30	25 s
**Wash**	1	DMF	2 mL	30	1 min

*Time required to reach the desired temperature.

Module E1: Fmoc deprotection - piperidine (7 min)

The resin rested in DMF (3 mL) until the reaction vessel was adjusted to 25 °C, then it was washed with DMF (3 x 2 mL for 15 s). Fmoc deprotection solution A (2 mL) was delivered to the reaction vessel. After 5 min, the reaction solution was drained, and the resin washed with DMF (3 mL for

15 s) and CH_2Cl_2 (3 x 3 mL for 30 s each). The temperature of the reaction vessel was decreased for the next module.

Action	Cycles	Solution	Amount	T (°C)	Incubation time
**Wash**	3	DMF	2 mL	25	15 s
**Delivery**	1	Fmoc depr. Solution A	2 mL	25	5 min
**Wash**	1	DMF	3 mL	25	15 s
**Wash**	3	CH_2Cl_2	3 mL	25	30 s
**Cooling**	-	-	-	T_1	-

Module E2: Lev deprotection (35 min)

The resin was washed with CH_2Cl_2 (3 x 3 mL for 15 s), and the temperature was adjusted to 35°C. CH_2Cl_2 (2 mL) was delivered to dilute Lev deprotection solution (1 mL). The reagents were kept under pulsed argon bubbling for 10 min and washed three with CH_2Cl_2 (3 x 3 mL for 15 s). This procedure was repeated three times (highlighted in green). The reaction solution was drained, and the resin was washed with DMF (3 x 3 mL for 30 s) and CH_2Cl_2 (3 x 2 mL for 30 s). The temperature of the reaction vessel was decreased for the next module.

Action	Cycles	Solution	Amount	T (°C)	Incubation time
**Wash**	3	DMF	3 mL	35	15 s
**Delivery**	1	CH_2Cl_2	2 mL	35	-
**Delivery**	1	Lev depr. solution	1 mL	35	10 min
**Wash**	3	CH_2Cl_2	3 mL	35	15 s
**Wash**	3	DMF	3 mL	35	30 s
**Wash**	3	CH_2Cl_2	2 mL	35	30 s
**Cooling**	-	-	-	T_1	-

d. Post-synthesizer manipulations (Post-AGA).

Module F: On-resin methanolysis

The resin was suspended in a THF: MeOH solution (4:1, 4 mL), and sodium methoxide in methanol (0.5 M, 400 µL) was added. The mixture was gently shaken at room temperature. After microcleavage (see Module H) indicated the complete hydrolysis of all ester groups, the resin was repeatedly washed with THF (3 x 4 mL), MeOH (3 x 4 mL), and CH_2Cl_2 (3 x 4 mL). The reaction time is variable, and is indicated for each synthesis.

Module G: Cleavage from solid support

As described previously, the oligosaccharides were cleaved from the solid support using a continuous-flow photoreactor.[3] CH_2Cl_2 was utilized as the carrier solvent system (15% MeOH was added for sulfated compounds).

Module H: Micro-cleavage from solid support

Trace amounts of resin (around 20 beads) was dispersed in CH_2Cl_2 (0.1 mL) and irradiated with an UV lamp (6 W, 356 nm) for 20 min. Acetonitrile was then added to the resin, and the resulting solution was analyzed by MALDI.

Module I: Hydrogenolysis[a]

The crude compound was dissolved in solvent (see below for specific conditions). 10% Pd/C (3x w/w residue) was added, and the reaction was stirred in a vial equipped with a H_2 balloon. The reaction progress was monitored to avoid undesired side product formation (by ESI-MS negative mode or MALDI). Upon completion, the reaction was filtered by a PTFE syringe filter and washed with H_2O. The filtrate was concentrated by lyophilization.

[a]Reaction times and solvents are indicated for each synthesis. THF must be inhibitor-free if used as the solvent.

Module J: Purification/Analysis

Purification of the crude products was achieved using a manual size-exclusion column or reverse phase HPLC (Agilent 1200 Series). The pure compounds were analyzed using analytical HPLC (Agilent 1200 Series)

- **Method J_{1P}:** (Hypercarb column, 150 x 10 mm) flow rate of 3.0 mL/min with H_2O (0.1% formic acid) and ACN as eluents [isocratic (5 min), linear gradient to 20% ACN (40 min), linear gradient to 100% ACN (10 min), isocratic 100% ACN (10 min)]. ELSD Detector: 60 °C.
- **Method J_{2P}:** (Synergi Hydro RP18 column, Phenomenex, 250 x 10 mm) flow rate of 4.0 mL/min with H_2O (0.1% formic acid) and ACN as eluents [isocratic (5 min), linear gradient to 30% ACN (30 min), linear gradient to 100% ACN (10 min), isocratic 100% ACN (5 min)]. ELSD Detector: 60 °C.
- **Method J_{1A}:** (Synergi Hydro RP18 column, Phenomenex, 250 x 4.6 mm), flow rate of 1.0 mL/min with H_2O (0.1% formic acid) and ACN as eluents [isocratic (5 min), linear gradient to 70% ACN (30 min), linear gradient to 100% ACN (10 min), isocratic 100% ACN (5 min)]. ELSD Detector: 60 °C.
- **Method J_{2A}:** (Hypercarb column, 150 x 4.60 mm) flow rate of 0.7 mL/min with H_2O (0.1% formic acid) and ACN as eluents [isocratic (5 min), linear gradient to 70% ACN (30 min), linear gradient to 100% ACN (10 min), isocratic 100% ACN (5 min)]. ELSD Detector: 60 °C.

e. Building blocks for AGA.

All Building blocks (Figure S 1) were purchased from GlycoUniverse GmbH.

Merrifield resin equipped with a photocleavable linker **1** (loading 0.45 mmol/g) was prepared according to literature procedures (3). AGA syntheses were performed on a 0.015 mmol scale.

f. Oligosaccharides syntheses.

Figure S 2 shows the synthetizes glycans.

i. Synthesis of M8.

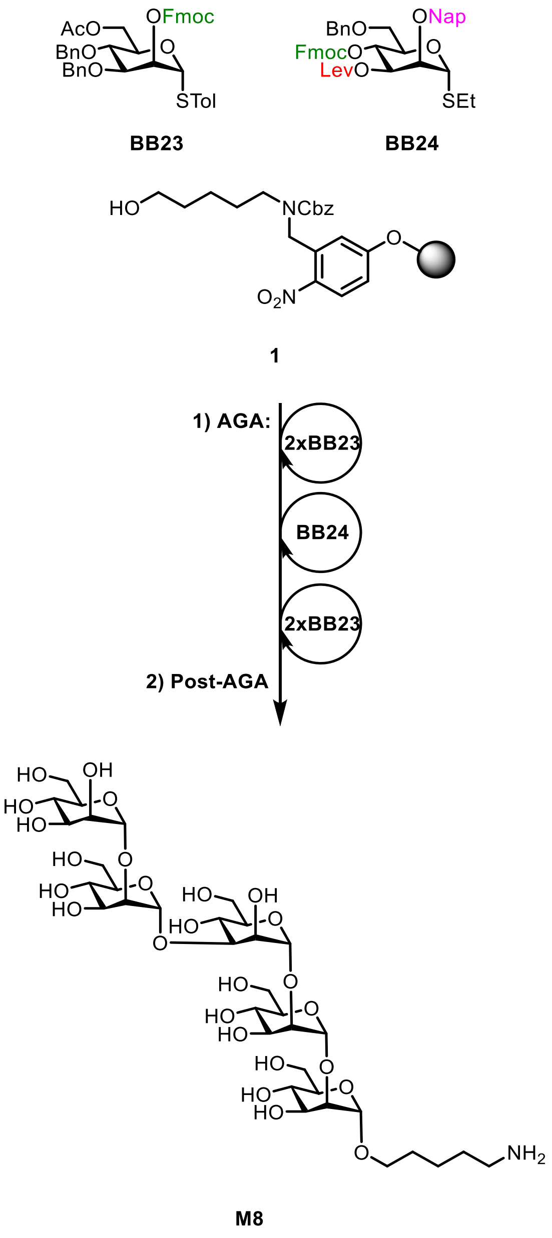

Step		Modules	Notes
AGA		**A**	
	**2x BB23**	**B, C, D, E1**	**C:** (-30 °C for 10 min, -5 °C for 20 min)
	**BB24**	**B, C, D, E2**	**C:** (-20 °C for 10 min, 0 °C for 35 min)
	**2x BB23**	**B, C, D, E1**	**C:** (-30 °C for 10 min, -5 °C for 20 min)
Post-AGA	**Methanolysis**	**F**	**F:** 18 h
	**Photocleavage**	**G**	
	**Hydrogenolysis**	**I**	**I:** 18 h (2 mL, 2:1:1 EtOAc:H_2O:*t*BuOH)
	**Purification**	**J_{2P}**	

Compound **M8** was obtained as a white solid (0.85 mg, 6% overall yield).

Analytical data for **M8**:

^{1}H NMR (400 MHz, D_2O) δ 5.26 (s, 1H), 5.14 (s, 1H), 4.95 (s, 1H), 4.88 (dd, J = 7.7, 1.9 Hz, 2H), 4.10 – 3.33 (m, 32H), 2.91 – 2.77 (m, 2H), 1.60 – 1.44 (m, 4H), 1.36 – 1.20 (m, 2H).

^{13}C NMR (101 MHz, D_2O) δ 102.2, 100.7, 100.6, 98.0, 78.9, 78.6, 78.5, 73.3, 72.8, 70.2, 69.9, 69.9, 69.6, 67.7, 67.0, 66.9, 66.4, 61.1, 39.3, 28.0, 26.7, 22.5.

HRMS (QToF): Calculated for $C_{35}H_{63}NO_{26}$ $[M+H]^+$ 914.3711 measured 914.3774

^{1}H NMR of M8 (400 MHz, D_2O)

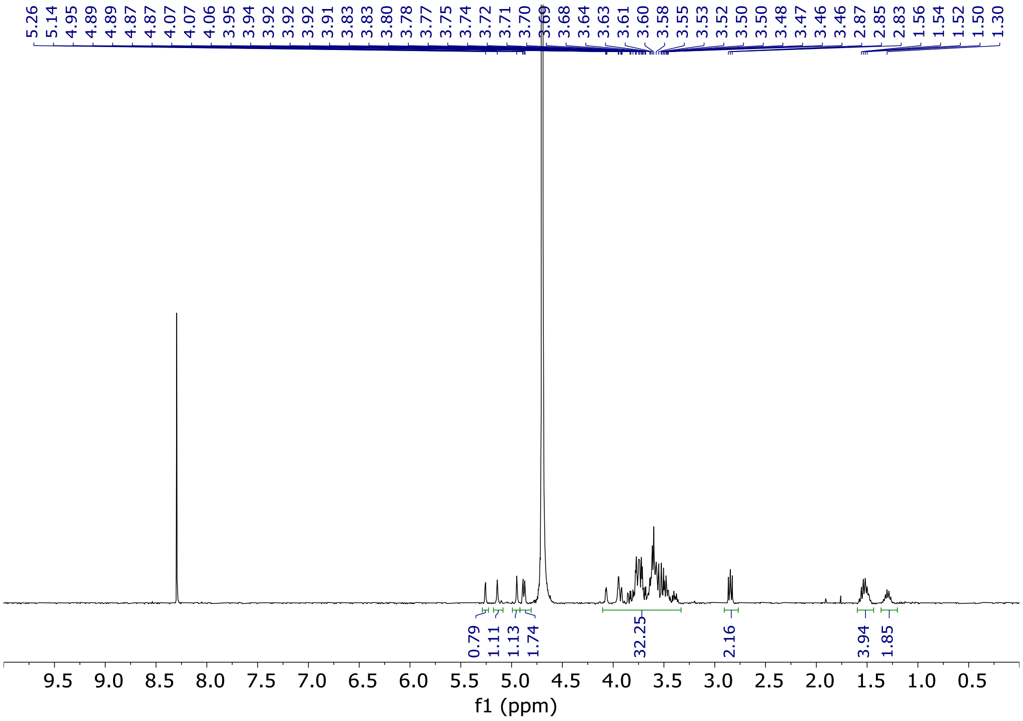

^{1}H-^{1}H COSY NMR of M8 (D_2O)

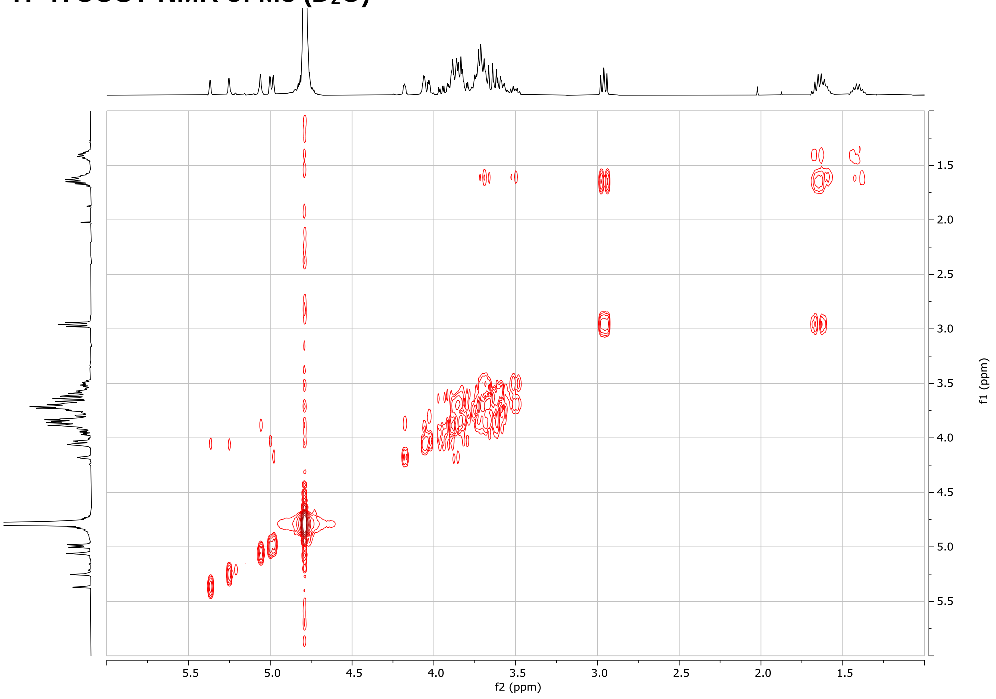

^{1}H-^{13}C HSQC NMR of M8 (D_2O)

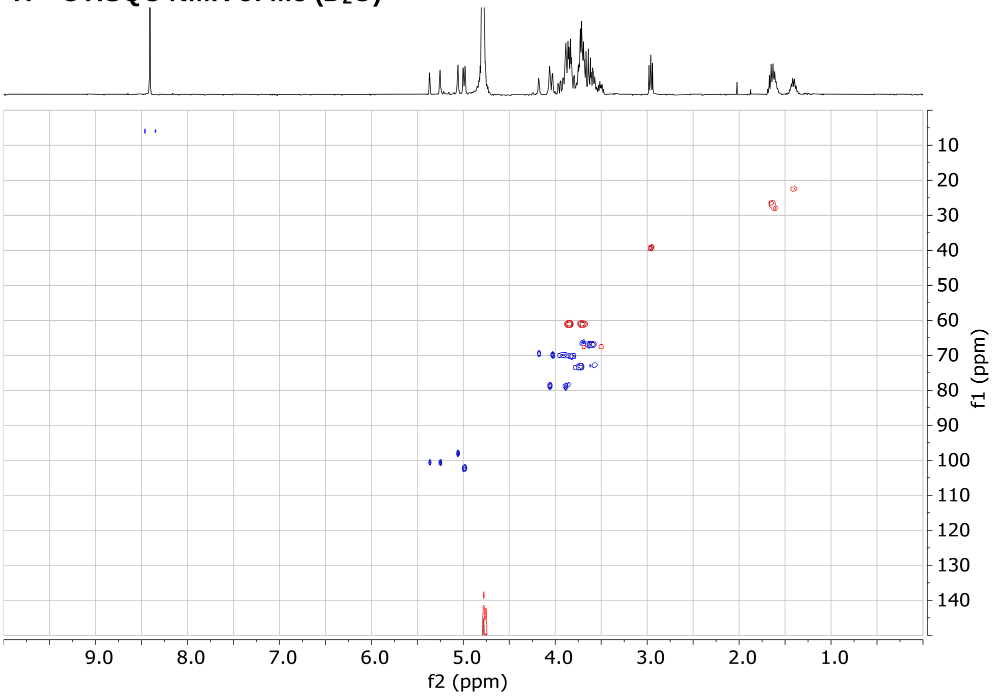

RP-HPLC of M8 (ELSD trace, Method J_{2A}, t_R= 26.18 min)

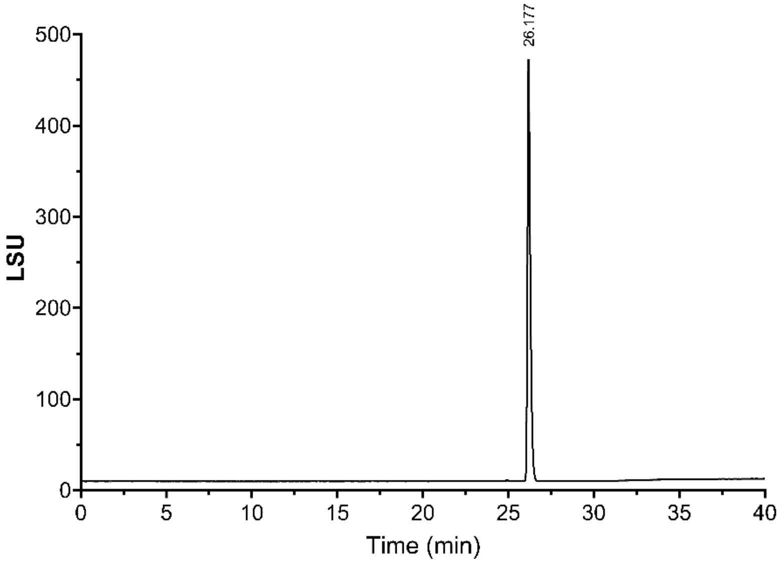

ii. Synthesis of G1

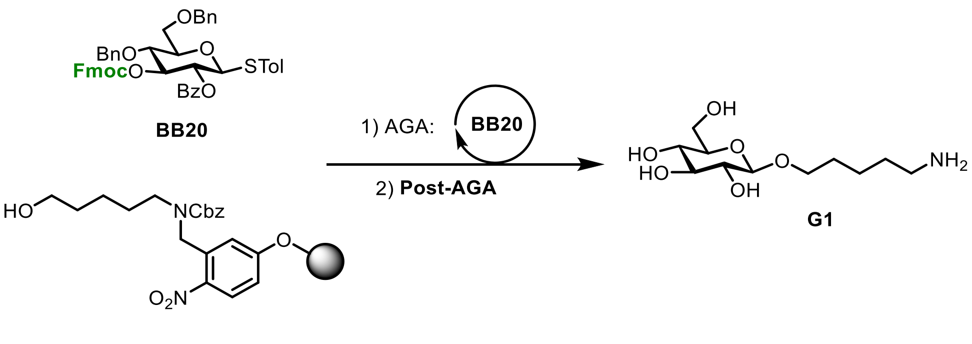

Step		Modules	Notes
AGA		**A**	
	**BB20**	**B, C, D, E1**	**C:** (-20 °C for 15 min, 0 °C for 30 min)
Post-AGA	**Methanolysis**	**F**	**F:** 18 h
	**Photocleavage**	**G**	
	**Hydrogenolysis**	**I**	**I:** 18 h (2 mL, 2:1:1 EtOAc:H_2O:*t*BuOH)
	**Purification**	**J_{1P}**	

Compound **G1** was obtained as a white solid (0.6 mg, 15% overall yield).

Analytical data for **G1**:

^{1}H NMR (400 MHz, D_2O) δ 4.42 (d, *J* = 8.0 Hz, 1H), 3.95 – 3.83 (m, 2H), 3.74 – 3.59 (m, 2H), 3.50 – 3.29 (m, 3H), 3.22 (dd, *J* = 9.3, 8.0 Hz, 1H), 3.02 – 2.91 (t, *J* = 7.4 Hz, 1H), 1.72 – 1.58 (m, 4H), 1.43 (m, 2H).

^{13}C NMR (101 MHz, D_2O) δ 102.3, 76.0, 75.9, 75.9, 75.9, 73.1, 70.1, 70.1, 69.9, 69.8, 60.9, 60.7, 60.7, 39.3, 28.3, 26.4, 22.0

HRMS (QToF): Calculated for $C_{11}H_{24}NO_6$ $[M+H]^+$ 266.1598; found 266.1609.

^{1}H NMR of G1 (400 MHz, D_2O)

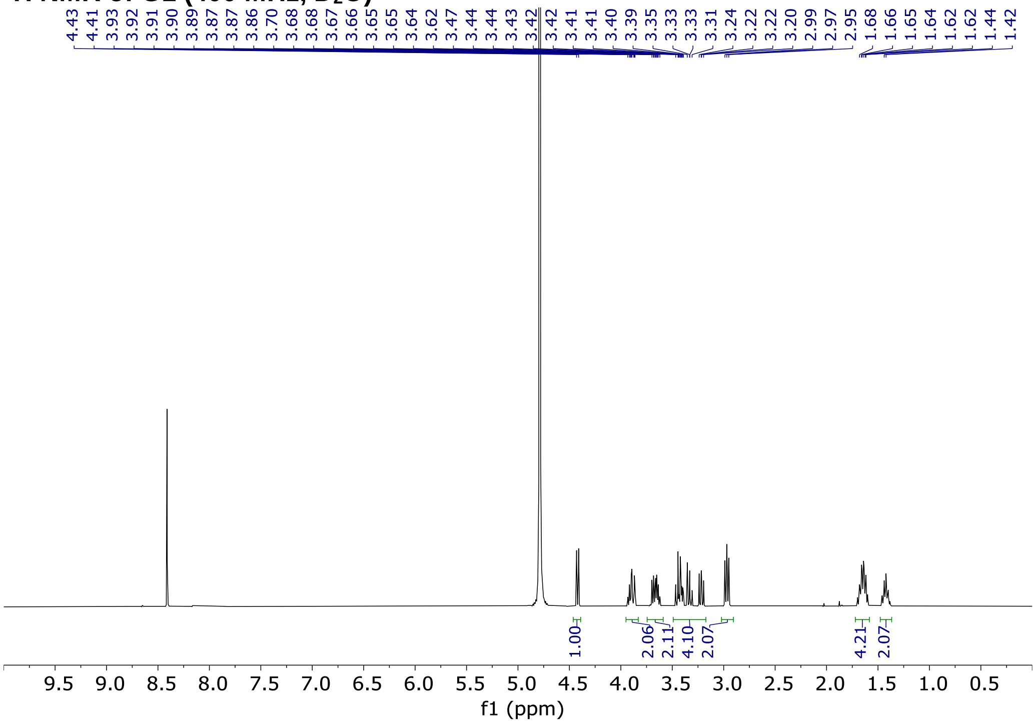

^{1}H-^{1}H COSY NMR of G1 (D_2O)

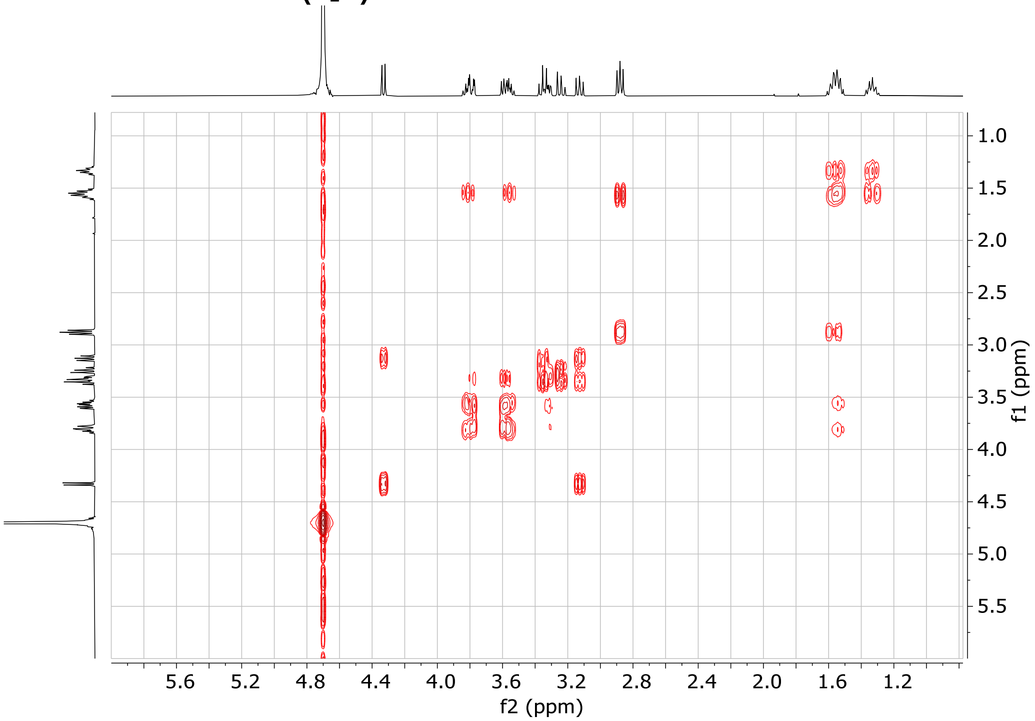

^{1}H-^{13}C HSQC NMR of G1 (D_2O)

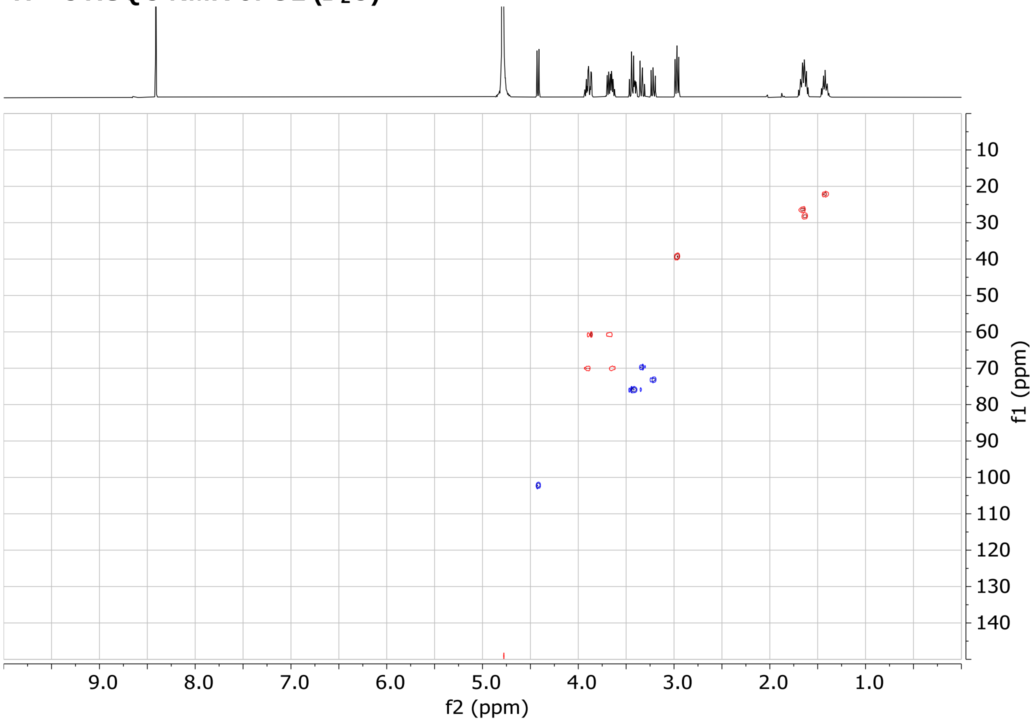

RP-HPLC of G1 (ELSD trace, Method J_{1A}, t_R= 12.35 min)

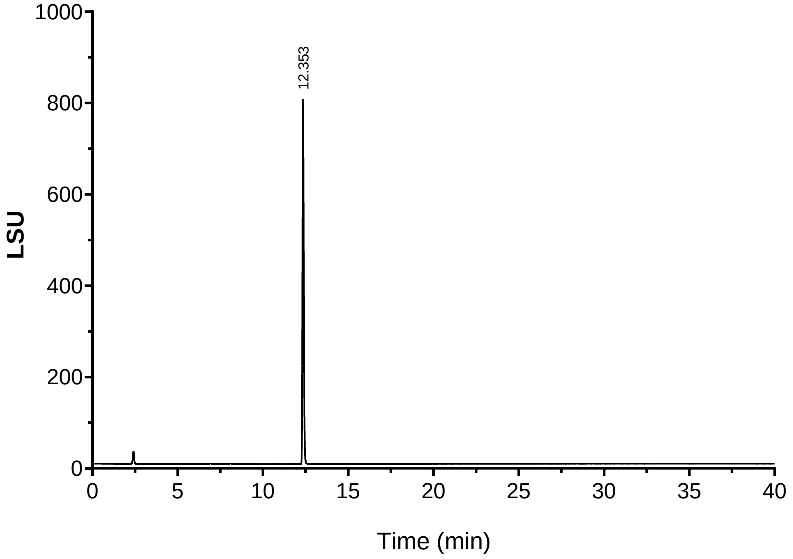

iii. Synthesis of G2.

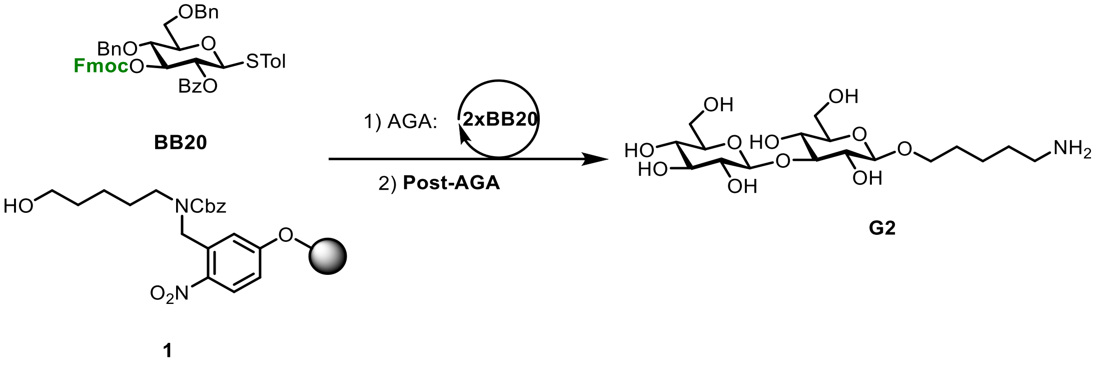

Step		Modules	Notes
AGA		**A**	
	**2xBB20**	**B, C, D, E1**	**C:** (-20 °C for 15 min, 0 °C for 30 min)
Post-AGA	**Methanolysis**	**F**	**F:** 18 h
	**Photocleavage**	**G**	
	**Hydrogenolysis**	**I**	**I:** 18 h (2 mL, 2:1:1 EtOAc:H_2O:*t*BuOH)
	**Purification**	**J_{1P}**	

Compound **G2** was obtained as a white solid (0.7 mg, 11% overall yield).

Analytical data for **G2**:

^{1}H NMR (400 MHz, D_2O) δ 4.69 (d, *J* = 7.9 Hz, 1H), 4.44 (d, *J* = 8.1 Hz, 1H), 3.95 – 3.82 (m, 3H), 3.73 – 3.61 (m, 4H), 3.49 – 3.27 (m, 7H), 2.96 (t, *J* = 7.5 Hz, 2H), 1.73 – 1.56 (m, 4H), 1.48 – 1.35 (m, 2H).

^{13}C NMR (101 MHz, D_2O) δ 102.7, 101.8, 84.3, 75.6, 73.4, 72.7, 70.0, 70.0, 69.5, 68.1, 60.6, 60.6, 60.6, 39.4, 28.1, 26.5, 22.1

HRMS (QToF): Calculated for $C_{17}H_{34}NO_{11}$ $[M+H]^+$ 428.2126; found 428.2135.

^{1}H NMR of G2 (400 MHz, D_2O)

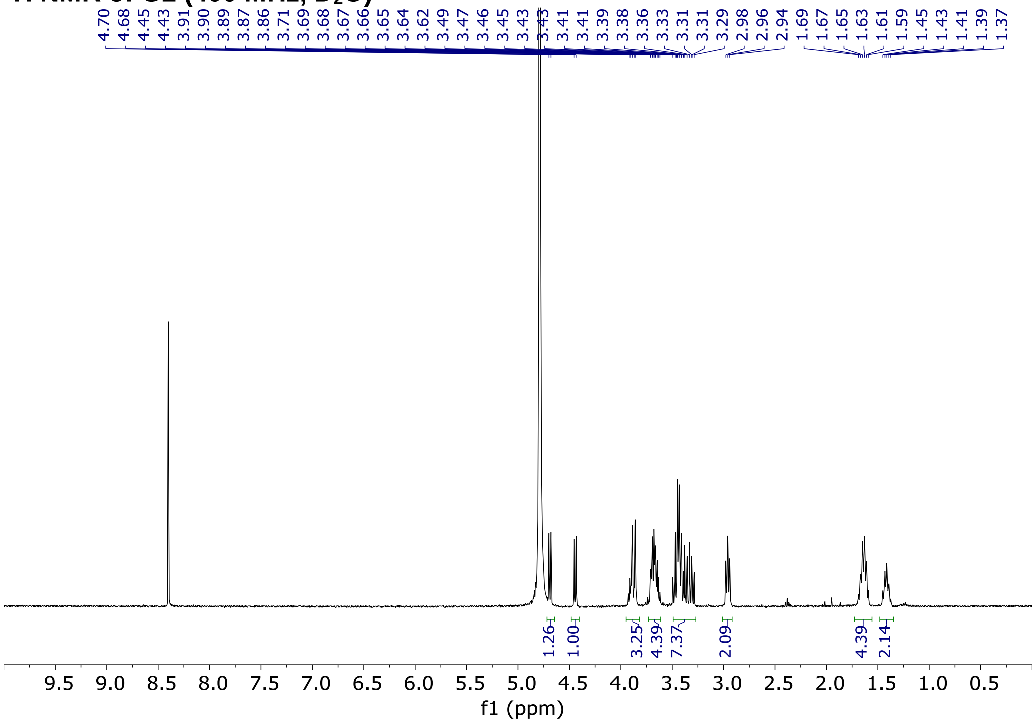

^{1}H-^{1}H COSY NMR of G2 (D_2O)

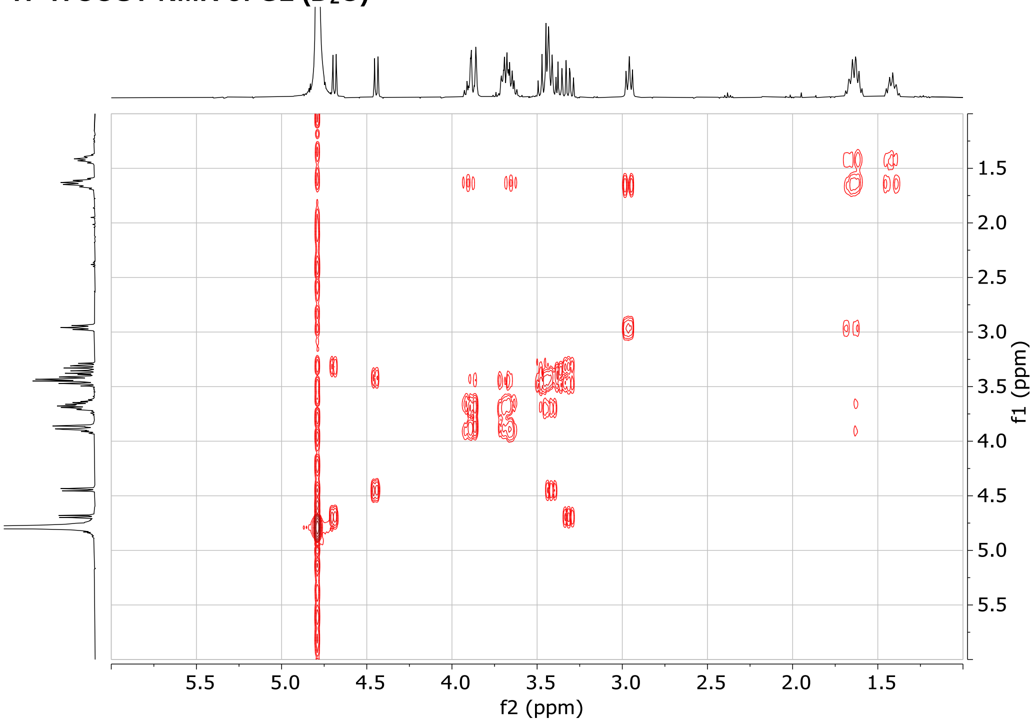

^{1}H-^{13}C HSQC NMR of G2 (D_2O)

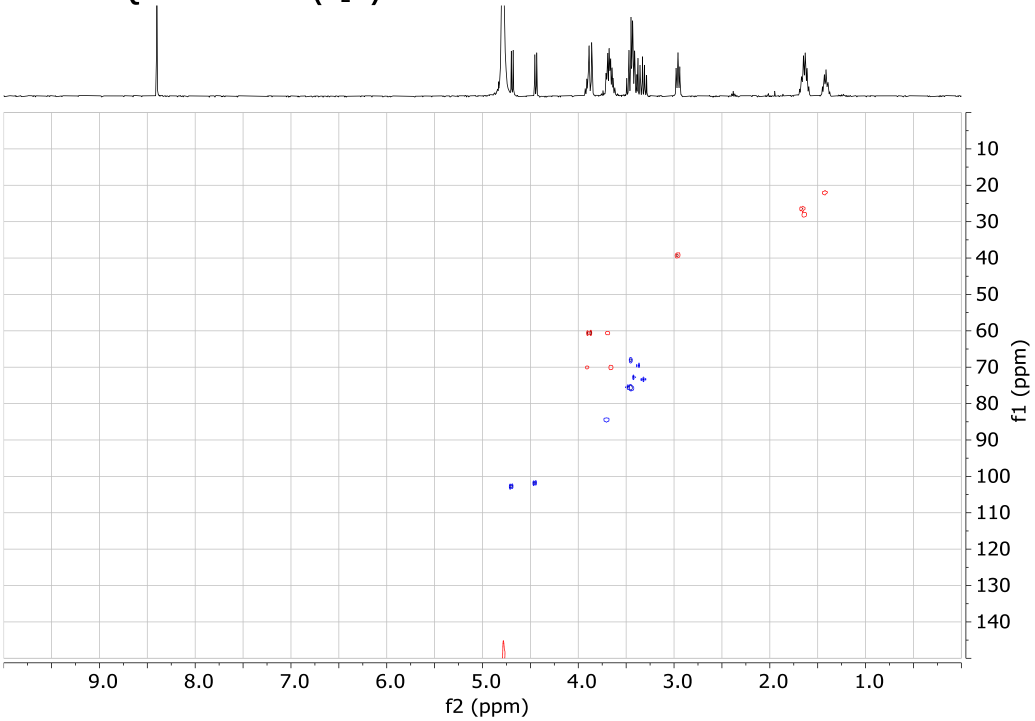

RP-HPLC of G2 (ELSD trace, Method J_{1A}, t_R= 11.95 min)

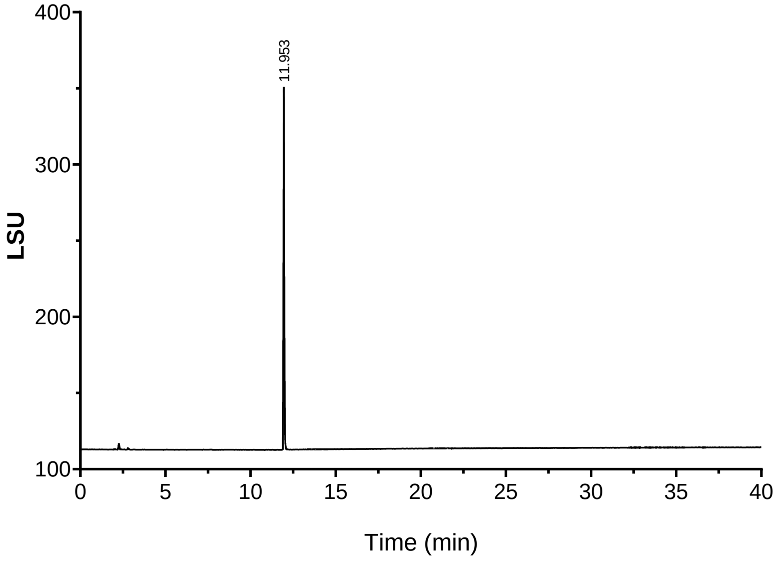

iv. Synthesis of G3.

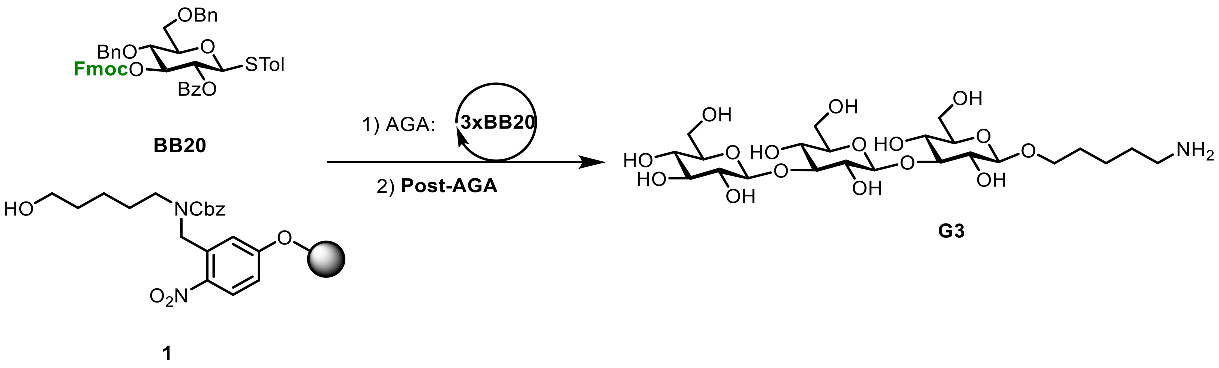

Step		Modules	Notes
AGA		**A**	
	**3xBB20**	**B, C, D, E1**	**C:** (-20 °C for 15 min, 0 °C for 30 min)
Post-AGA	**Methanolysis**	**F**	**F:** 18 h
	**Photocleavage**	**G**	
	**Hydrogenolysis**	**I**	**I:** 18 h (2 mL, 2:1:1 EtOAc:H_2O:*t*BuOH)
	**Purification**	**J_{1P}**	

Compound **G3** was obtained as a white solid (0.9 mg, 10% overall yield).

Analytical data for **G3**:

^{1}H NMR (400 MHz, D_2O) δ 4.73 (d, *J* = 8.0 Hz, 1H), 4.71 (d, *J* = 7.9 Hz, 1H), 4.44 (d, *J* = 8.1 Hz, 1H), 3.96 – 3.81 (m, 4H), 3.79 – 3.59 (m, 6H), 3.55 – 3.26 (m, 10H), 2.96 (t, *J* = 7.6 Hz, 2H), 1.75 – 1.57 (m, 4H), 1.48 – 1.35 (m, 2H).

^{13}C NMR (101 MHz, D_2O) δ 102.6, 101.8, 84.2, 75.6, 73.4, 73.2, 72.9, 70.0, 70.0, 69.5, 68.1, 60.6, 60.5, 39.2, 28.1, 26.3, 22.1

HRMS (QToF): Calculated for $C_{23}H_{44}NO_{16}$ $[M+H]^+$ 590.2655; found 590.2664.

^{1}H NMR of G3 (400 MHz, D_2O)

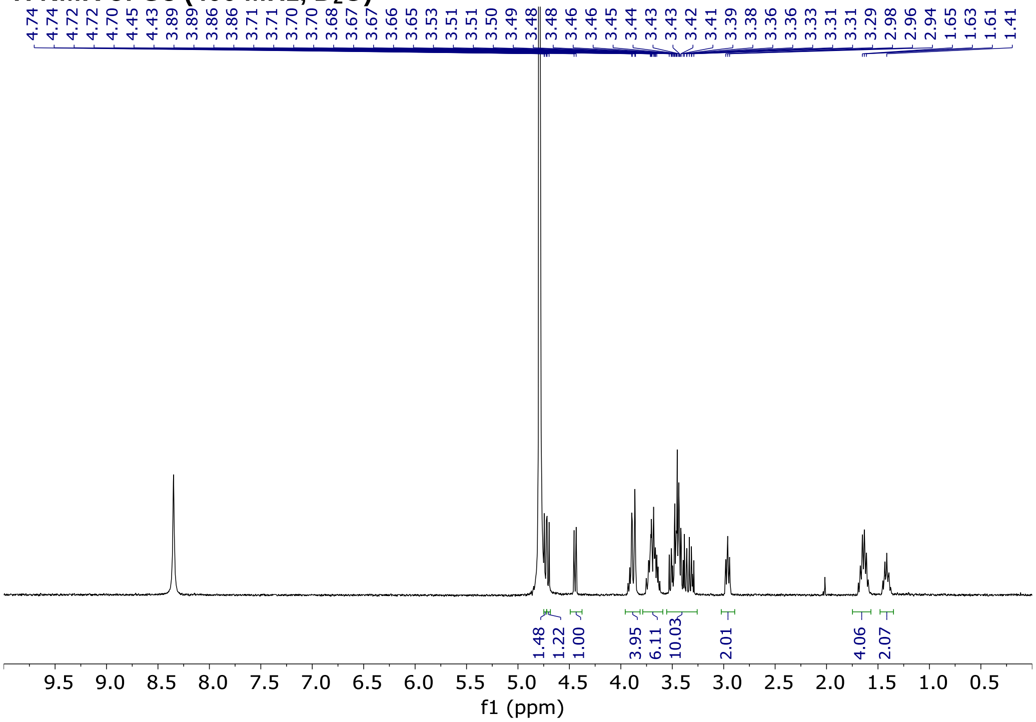

^{1}H-^{1}H COSY NMR of G3 (D_2O)

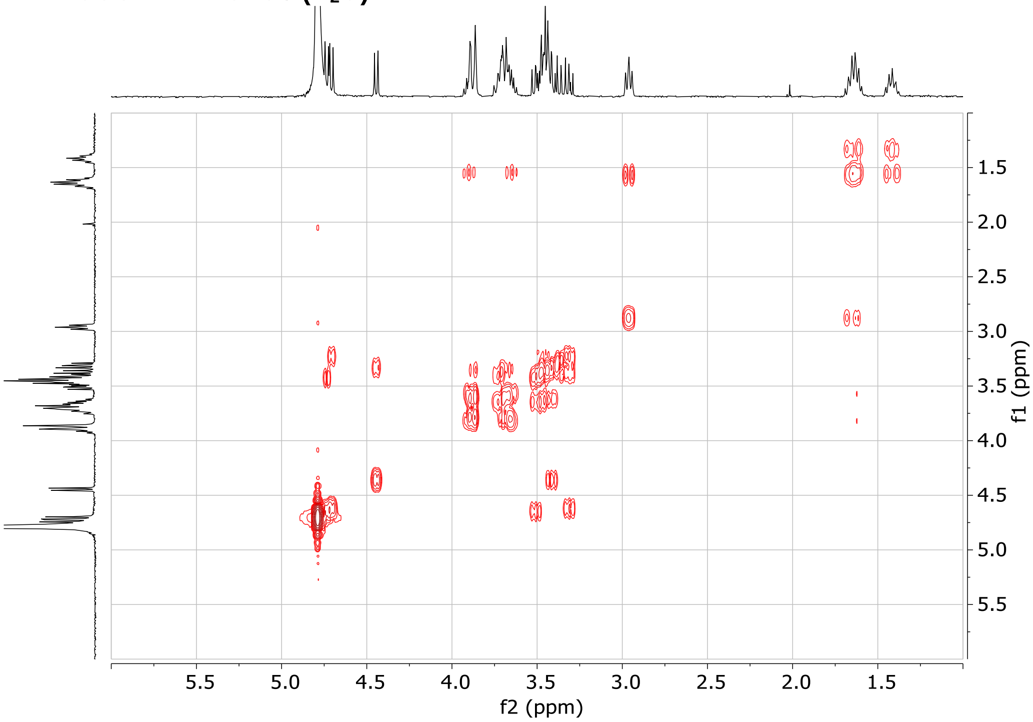

^{1}H-^{13}C HSQC NMR of G3 (D_2O)

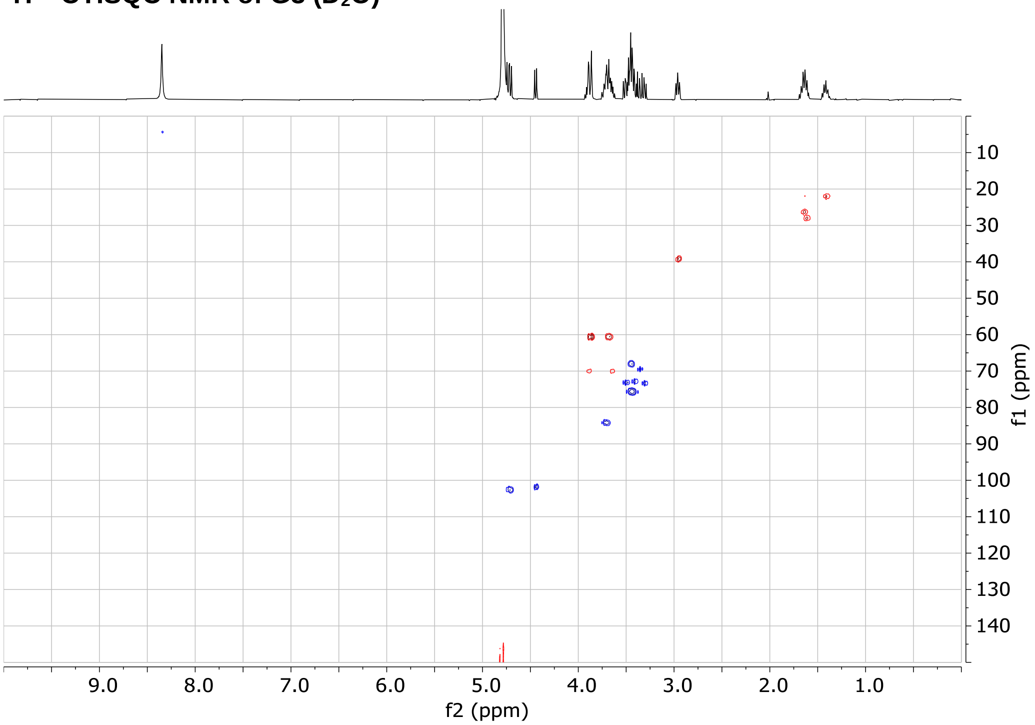

RP-HPLC of G3 (ELSD trace, Method J_{1A}, t_R= 12.20 min)

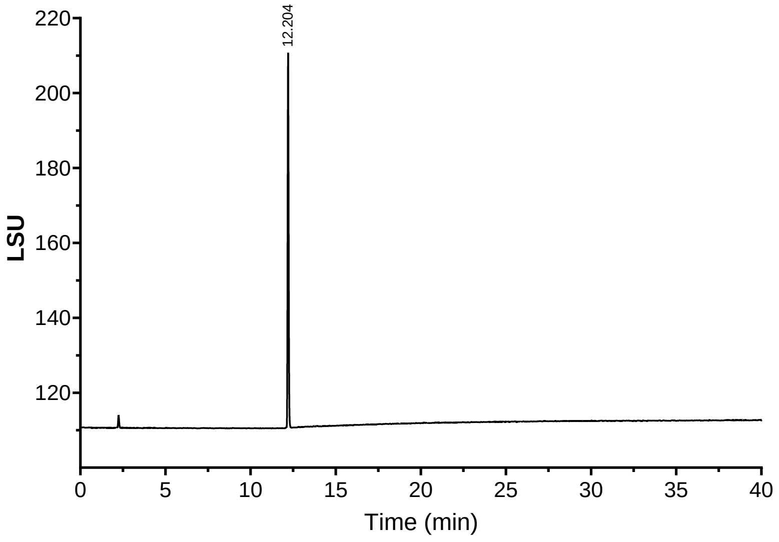

v. Synthesis of G5.

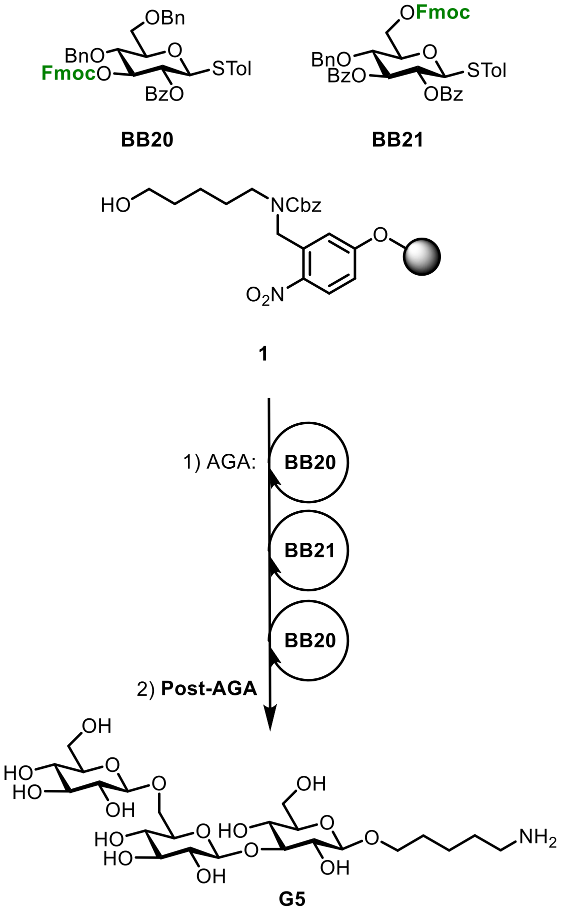

Step		Modules	Notes
AGA		**A**	
	**BB20**	**B, C, D, E1**	**C:** (-20 °C for 15 min, 0 °C for 30 min)
	**BB21**	**B, C, D, E2**	**C:** (-20 °C for 15 min, 0 °C for 30 min)
	**BB20**	**B, C, D, E1**	**C:** (-20 °C for 15 min, 0 °C for 30 min)
Post-AGA	**Methanolysis**	**F**	**F:** 18 h
	**Photocleavage**	**G**	
	**Hydrogenolysis**	**I**	**I:** 18 h (2 mL, 2:1:1 EtOAc:H_2O:*t*BuOH)
	**Purification**	**J_{1P}**	

Compound **G5** was obtained as a white solid (0.3 mg, 3% overall yield).

Analytical data for **G5**:

^{1}H NMR (400 MHz, D_2O) δ 4.66 (d, *J* = 7.9 Hz, 1H), 4.51 – 4.43 (m, 2H), 4.17 (d, *J* = 10.7 Hz, 1H), 3.96 – 3.78 (m, 4H), 3.77 – 3.55 (m, 5H), 3.55 – 3.21 (m, 10H), 2.96 (t, *J* = 7.5 Hz, 2H), 1.70 – 1.60 (m, 4H), 1.48 – 1.37 (m, 2H).

^{13}C NMR (101 MHz, D_2O) δ 102.9, 102.4, 101.9, 75.5, 74.7, 73.1, 72.6, 70.2, 70.0, 69.4, 68.7, 68.5, 68.2, 60.6, 60.6, 39.4, 28.1, 26.5, 22.0

HRMS (QToF): Calculated for $C_{23}H_{44}NO_{16}$ $[M+H]^+$ 590.2655; found 590.2667.

^{1}H NMR of G5 (400 MHz, D_2O)

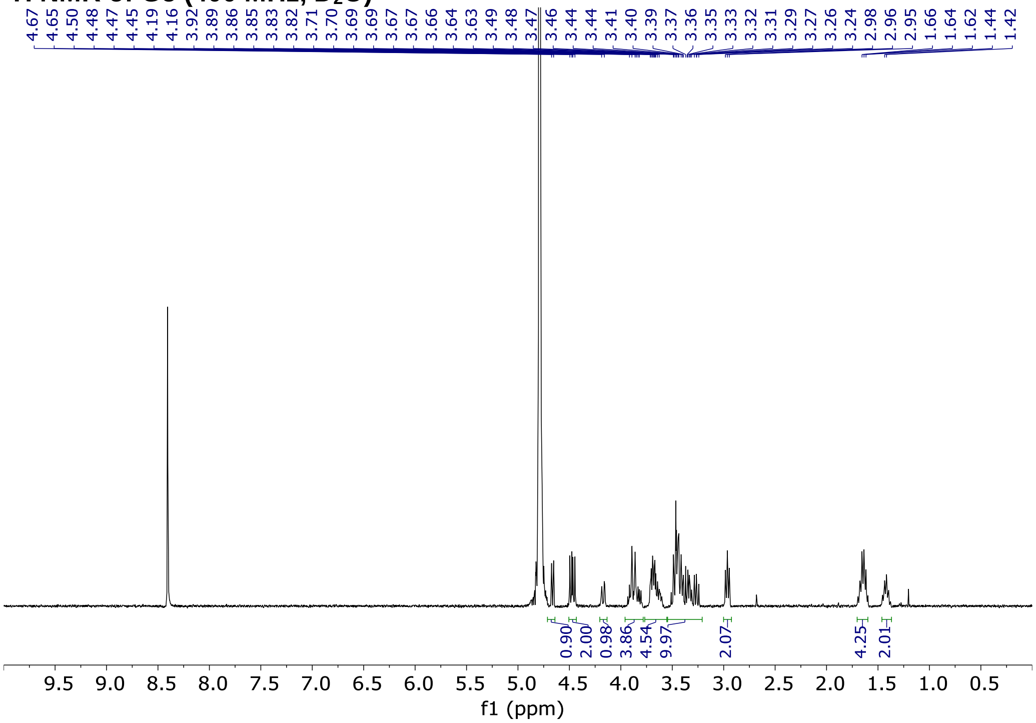

^{1}H-^{1}H COSY NMR of G5 (D_2O)

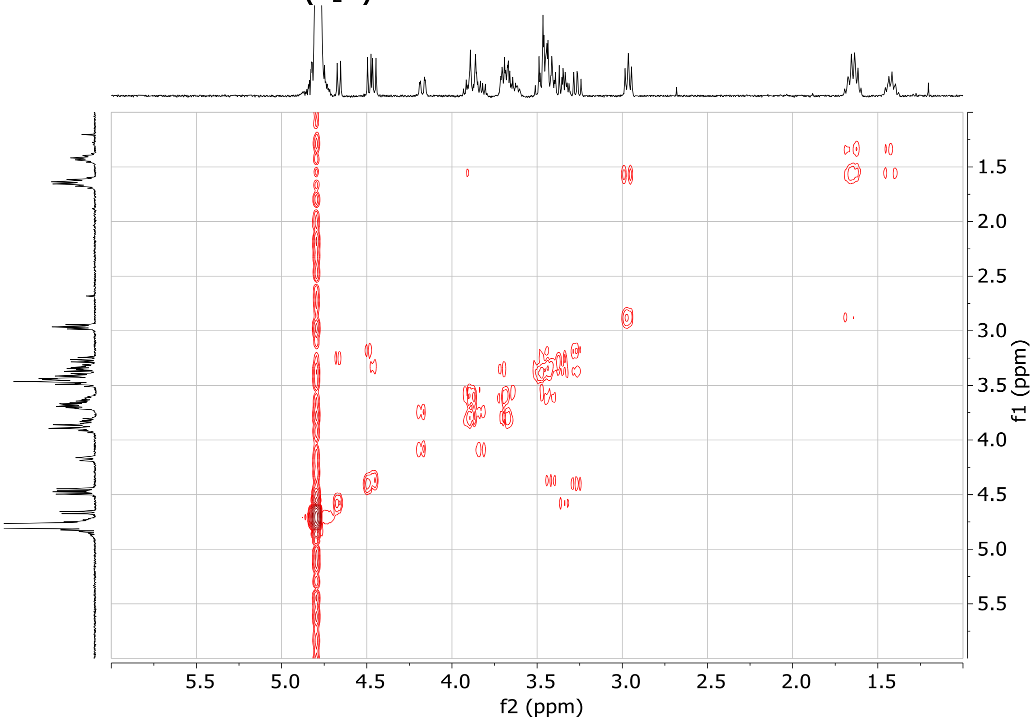

^{1}H-^{13}C HSQC NMR of G5 (D_2O)

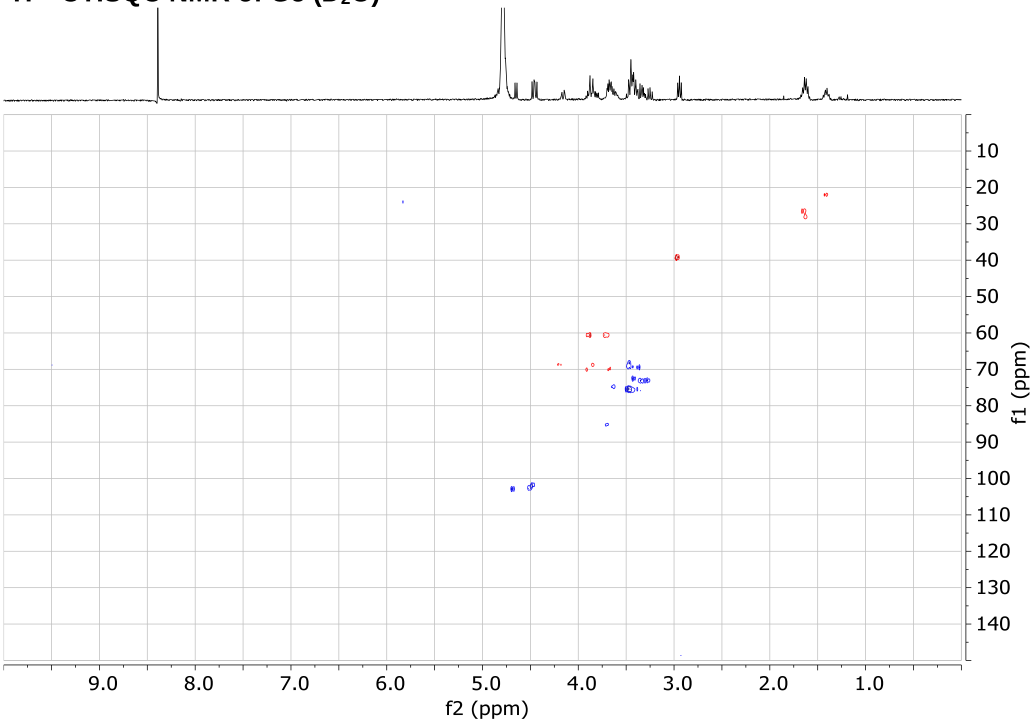

RP-HPLC of G5 (ELSD trace, Method J_{1A}, t_R= 12.25 min)

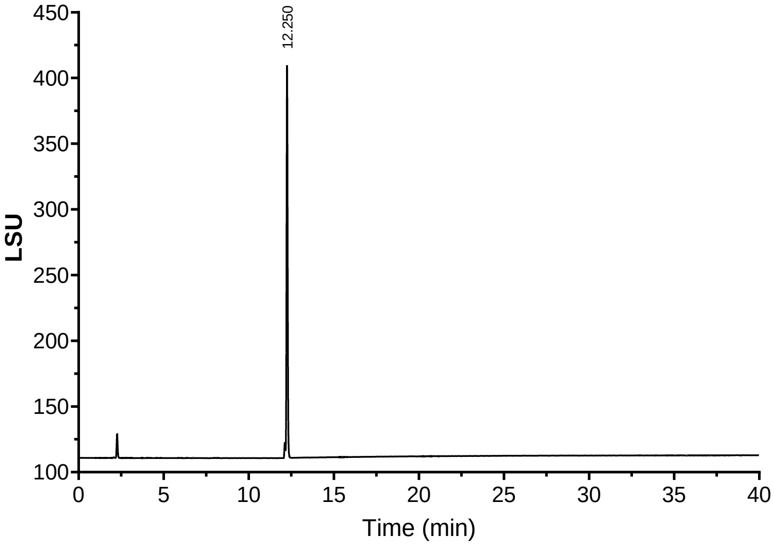

vi. Synthesis of G6.

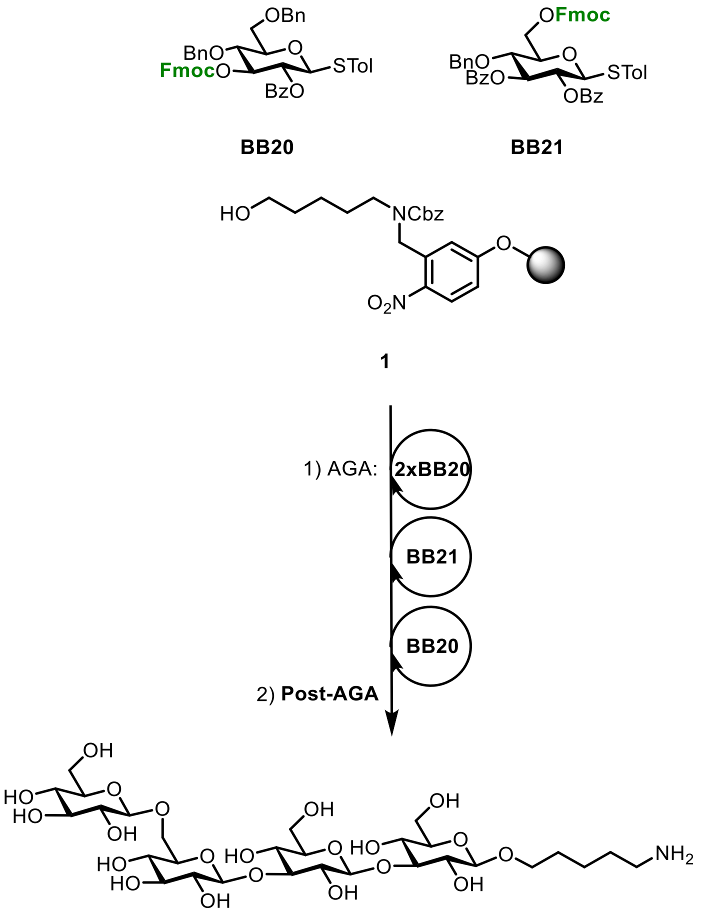

G6

Step		Modules	Notes
AGA		**A**	
	**2xBB20**	**B, C, D, E1**	**C:** (-20 °C for 15 min, 0 °C for 30 min)
	**BB21**	**B, C, D, E1**	**C:** (-20 °C for 15 min, 0 °C for 30 min)
	**BB20**	**B, C, D, E1**	**C:** (-20 °C for 15 min, 0 °C for 30 min)
Post-AGA	**Methanolysis**	**F**	**F:** 18 h
	**Photocleavage**	**G**	
	**Hydrogenolysis**	**I**	**I:** 18 h (2 mL, 2:1:1 EtOAc:H_2O:*t*BuOH)
	**Purification**	**J_{1P}**	

Compound **G6** was obtained as a white solid (0.7 mg, 6% overall yield).

Analytical data for **G6**:

^{1}H NMR (400 MHz, D_2O) δ 4.75 – 4.64 (m, 2H), 4.49 (d, *J* = 7.9 Hz, 1H), 4.45 (d, *J* = 8.1 Hz, 1H), 4.17 (d, *J* = 10.8 Hz, 1H), 3.94 – 3.80 (m, 5H), 3.75 – 3.59 (m, 7H), 3.56 – 3.21 (m, 13H), 2.96 (t, *J* = 7.5 Hz, 2H), 1.70 – 1.59 (m, 4H), 1.42 (m, 2H).

^{13}C NMR (101 MHz, D_2O) δ 102.7, 102.4, 102.4, 101.9, 84.3, 75.5, 74.7, 73.2, 73.1, 72.9, 72.7, 70.0, 69.8, 69.5, 68.5, 68.5, 68.1, 60.5, 60.5, 39.2, 27.9, 26.3, 22.0

HRMS (QToF): Calculated for $C_{29}H_{54}NO_{21}$ $[M+H]^+$ 752.3183; found 752.3193.

^{1}H NMR of G6 (400 MHz, D_2O)

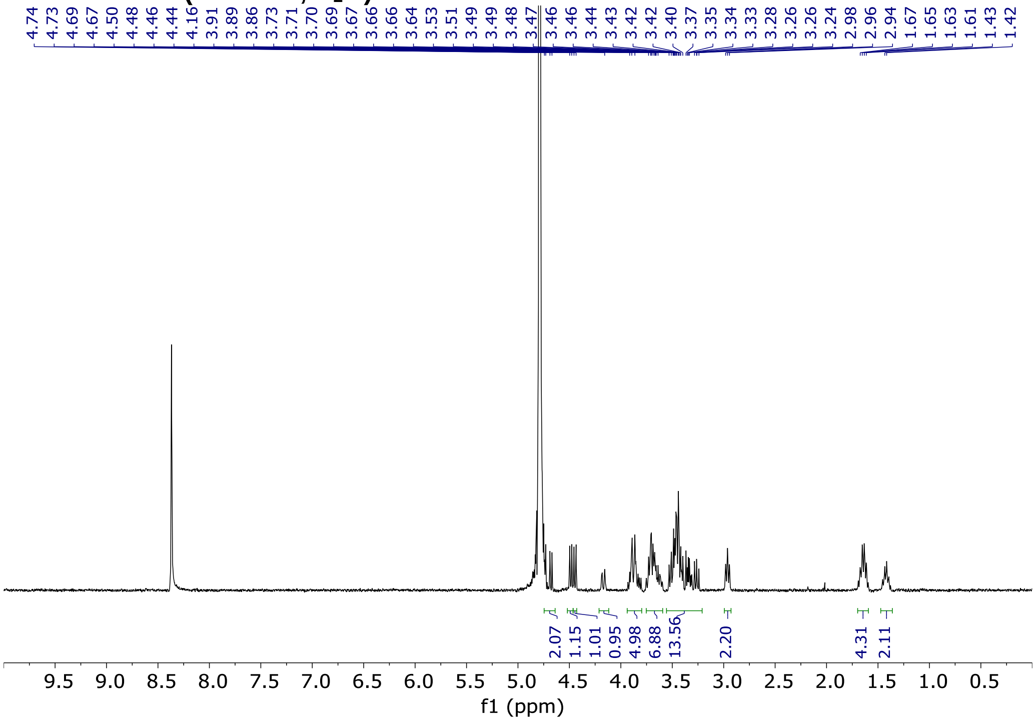

^{1}H-^{1}H COSY NMR of G6 (D_2O)

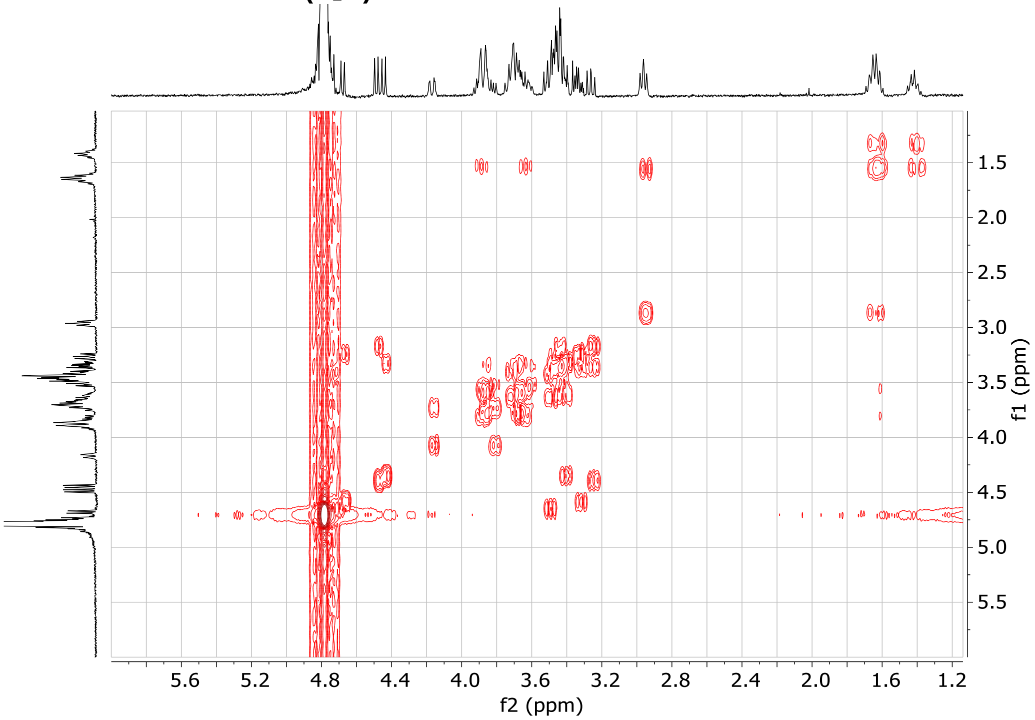

^{1}H-^{13}C HSQC NMR of G6 (D_2O)

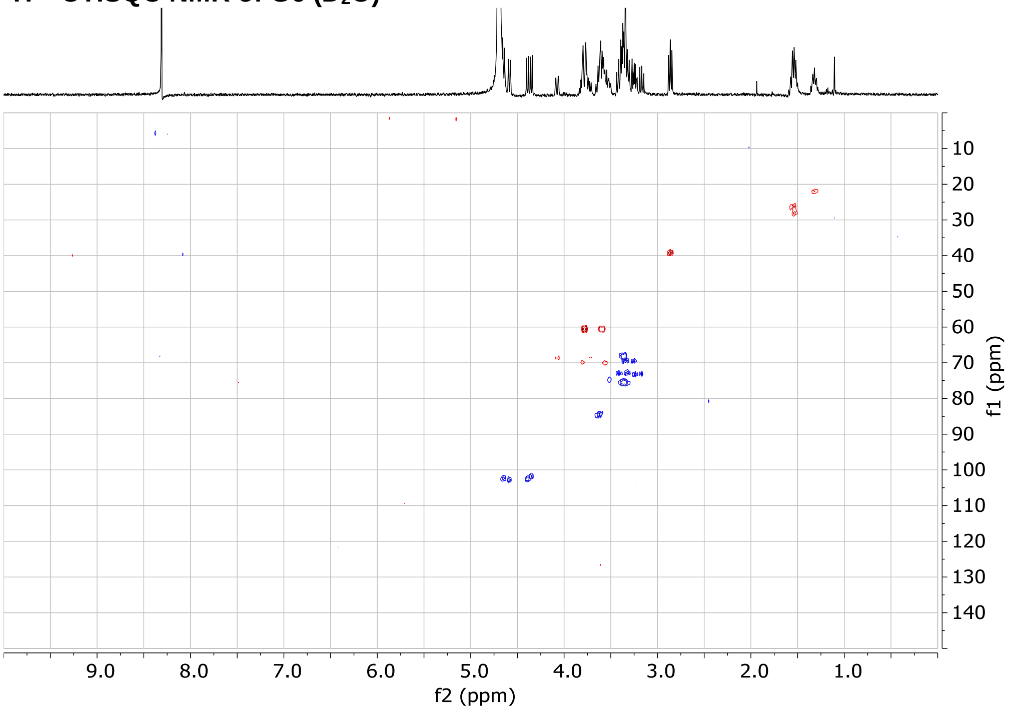

RP-HPLC of G6 (ELSD trace, Method J_{1A}, t_R= 12.43 min)

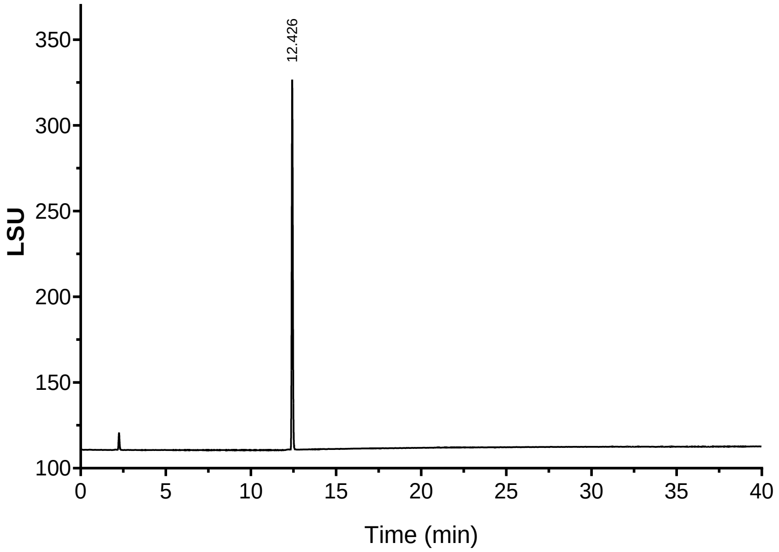

vii. Synthesis of G7.

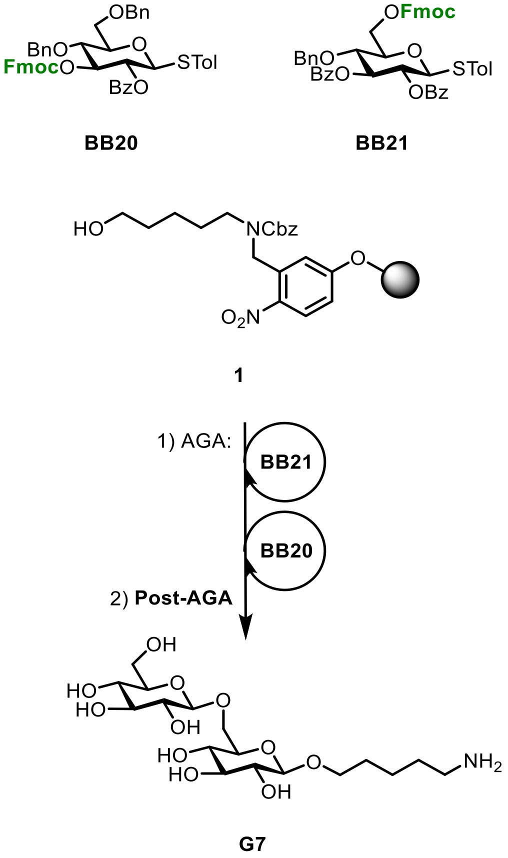

Step		Modules	Notes
AGA		**A**	
	**BB21**	**B, C, D, E1**	**C:** (-20 °C for 15 min, 0 °C for 30 min)
	**BB20**	**B, C, D, E1**	**C:** (-20 °C for 15 min, 0 °C for 30 min)
Post-AGA	**Methanolysis**	**F**	**F:** 18 h
	**Photocleavage**	**G**	
	**Hydrogenolysis**	**I**	**I:** 18 h (2 mL, 2:1:1 EtOAc:H_2O:*t*BuOH)
	**Purification**	$\mathbf{J_{1P}}$	

Compound **G7** was obtained as a white solid (0.6 mg, 9% overall yield).

Analytical data for **G7**:

^{1}H NMR (400 MHz, D_2O) δ 4.44 (dd, *J* = 17.4, 8.0 Hz, 2H), 4.15 (d, *J* = 11.6 Hz, 1H), 3.91 – 3.84 (m, 2H), 3.83 – 3.77 (m, 1H), 3.71 – 3.59 (m, 2H), 3.56 (t, *J* = 7.4 Hz, 1H), 3.46 – 3.18 (m, 7H), 2.95 (t, *J* = 7.5 Hz, 2H), 1.68 – 1.58 (m, 4H), 1.45 – 1.35 (m, 2H).

^{13}C NMR (101 MHz, D_2O) δ 102.7, 102.1, 75.6, 74.8, 73.1, 70.2, 70.2, 69.4, 68.5, 68.5, 60.6, 60.5, 39.2, 28.1, 26.3, 22.1

HRMS (QToF): Calculated for $C_{17}H_{34}NO_{11}$ $[M+H]^+$ 428.2126; found 428.2127.

^{1}H NMR of G7 (400 MHz, D_2O)

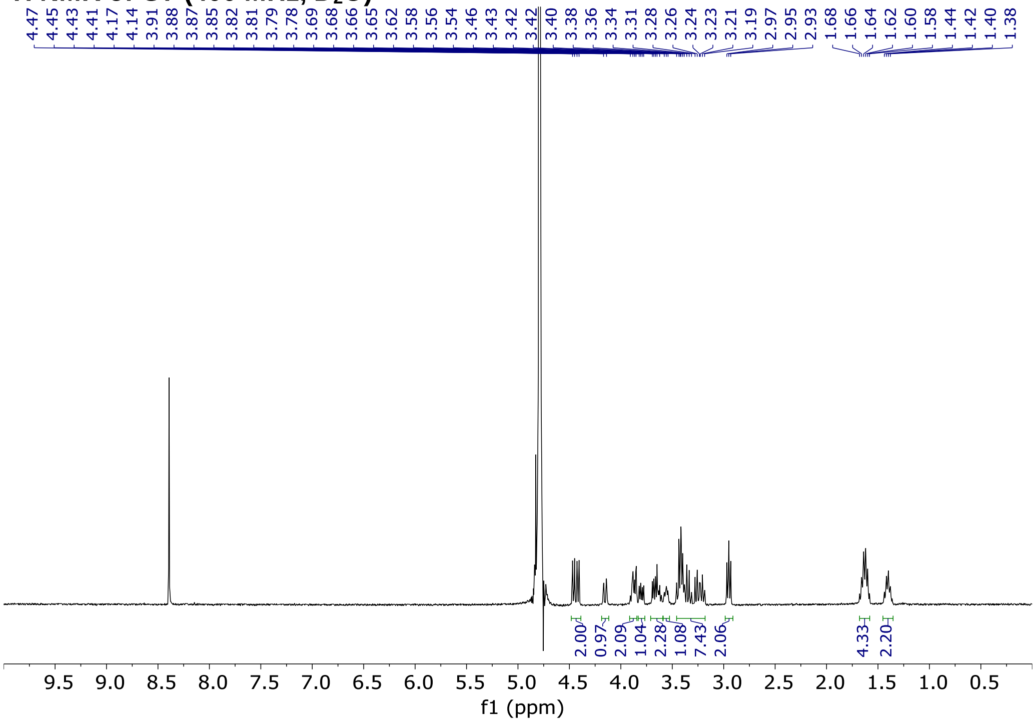

^{1}H-^{1}H COSY NMR of G7 (D_2O)

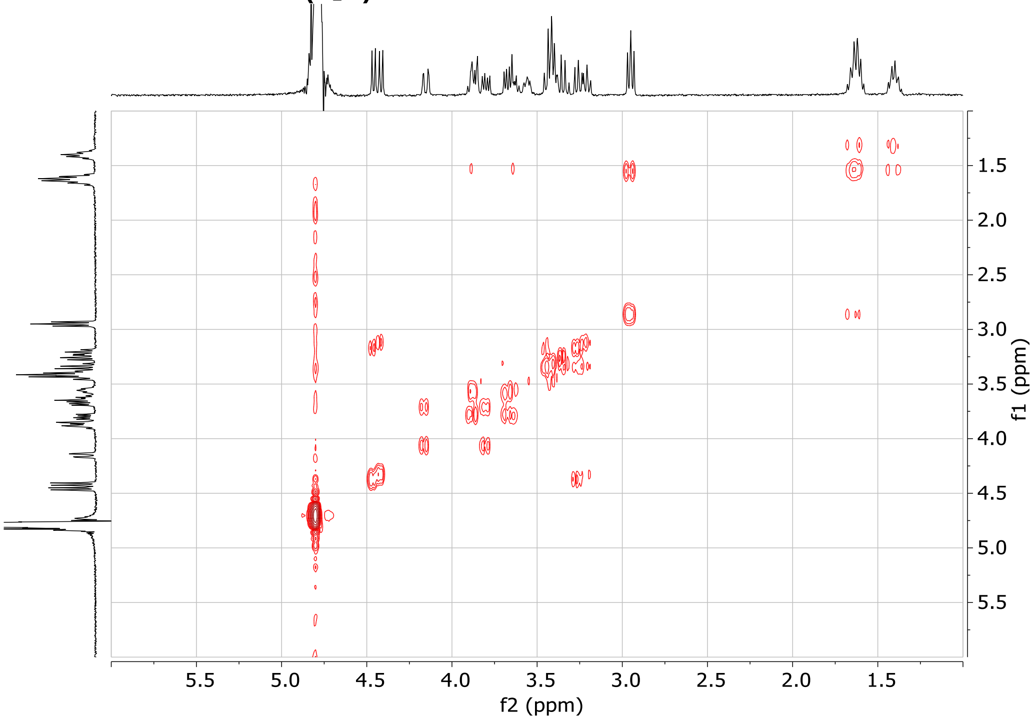

^{1}H-^{13}C HSQC NMR of G7 (D_2O)

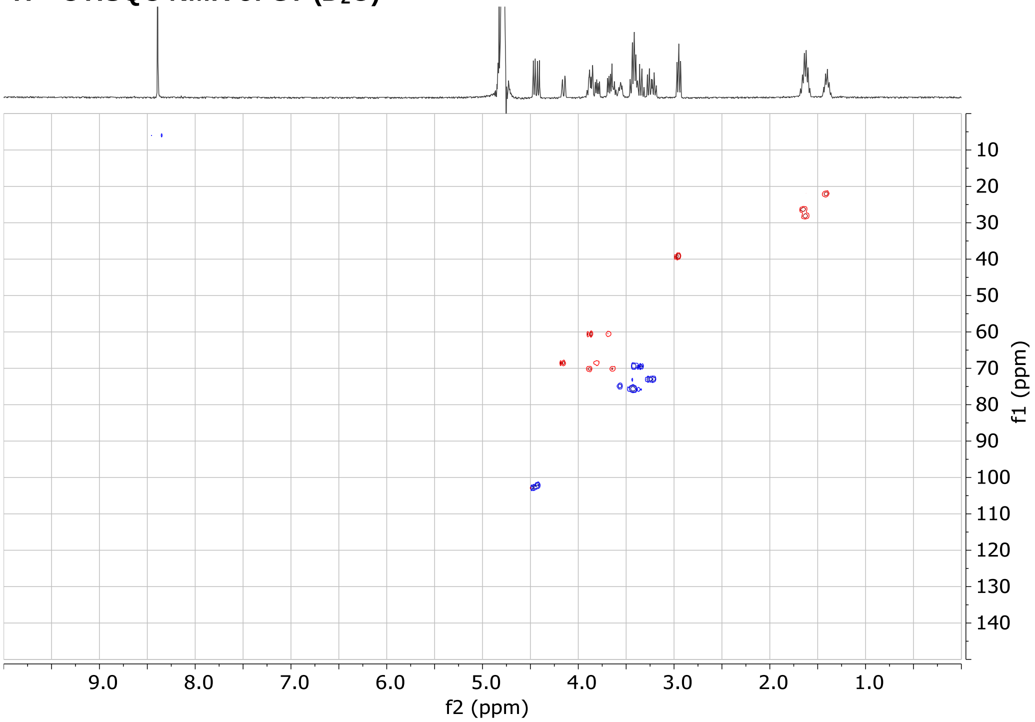

RP-HPLC of G7 (ELSD trace, Method J_{1A}, t_R= 11.99 min)

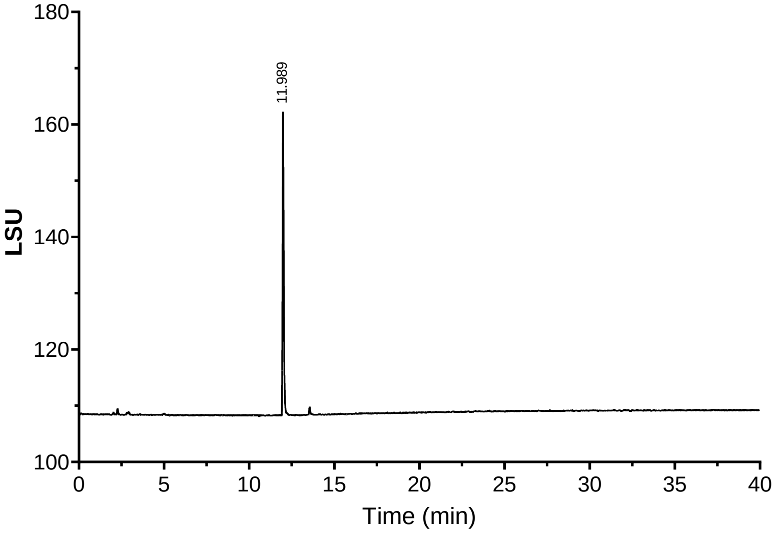

viii. Synthesis of G8.

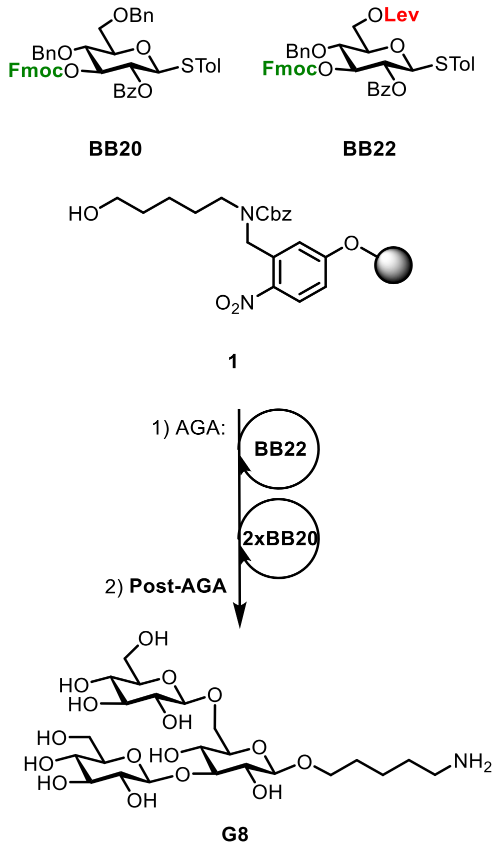

Step		Modules	Notes
AGA		**A**	
	**BB22**	**B, C, D, E2, E1**	**C:** (-20 °C for 15 min, 0 °C for 30 min)
	**2x BB20**	**B, C, D, E1**	**C:** (-20 °C for 15 min, 0 °C for 30 min)
Post-AGA	**Methanolysis**	**F**	**F:** 18 h
	**Photocleavage**	**G**	
	**Hydrogenolysis**	**I**	**I:** 18 h (2 mL, 2:1:1 EtOAc:H_2O:*t*BuOH)
	**Purification**	**J_{1P}**	

Compound **G8** was obtained as a white solid (0.3 mg, 3% overall yield).

Analytical data for **G8**:

^{1}H NMR (400 MHz, D_2O) δ 4.68 (d, *J* = 8.0 Hz, 1H), 4.49 – 4.40 (m, 2H), 4.16 (d, *J* = 11.6 Hz, 1H), 3.93 – 3.77 (m, 4H), 3.73 – 3.21 (m, 16H), 2.95 (t, *J* = 7.5 Hz, 2H), 1.68 – 1.57 (m, 4H), 1.45 – 1.33 (m, 2H).

^{13}C NMR (101 MHz, D_2O) δ 103.1, 102.7, 84.5, 75.6, 74.7, 73.5, 72.9, 70.5, 70.5, 69.7, 68.7, 68.7, 68.2, 60.8, 60.8, 39.4, 28.4, 26.5, 22.3

HRMS (QToF): Calculated for $C_{23}H_{44}NO_{16}$ $[M+H]^+$ 590.2655; found 590.2662.

^{1}H NMR of G8 (400 MHz, D_2O)

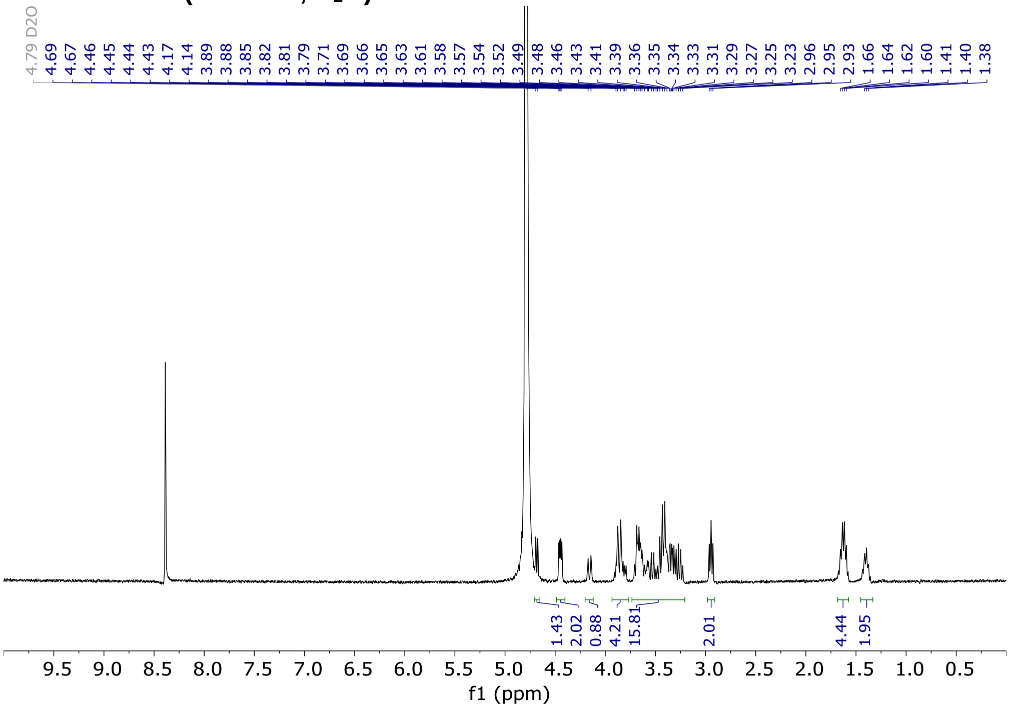

^{1}H-^{1}H COSY NMR of G8 (D_2O)

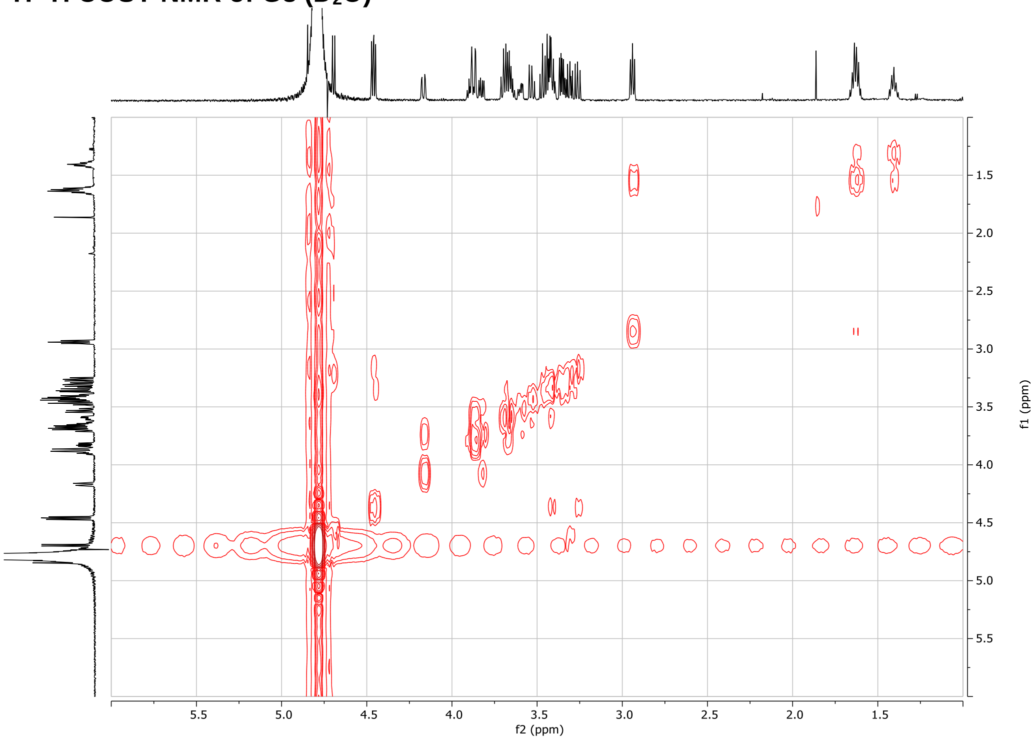

^{1}H-^{13}C HSQC NMR of G8 (D_2O)

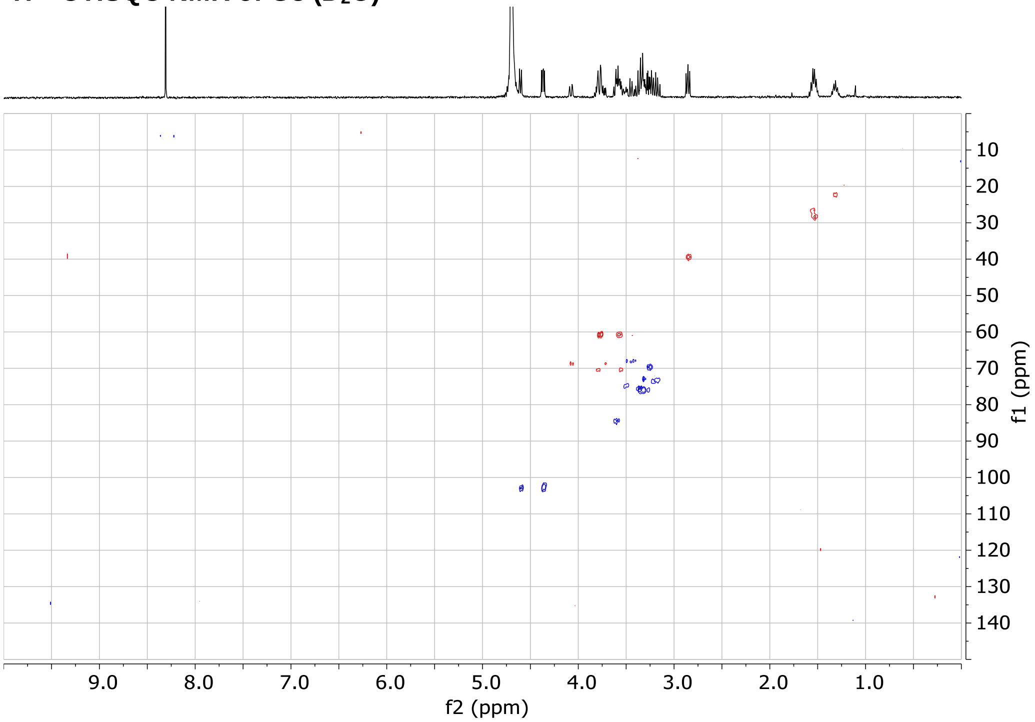

RP-HPLC of G8 (ELSD trace, Method J_{1A}, t_R= 12.16 min)

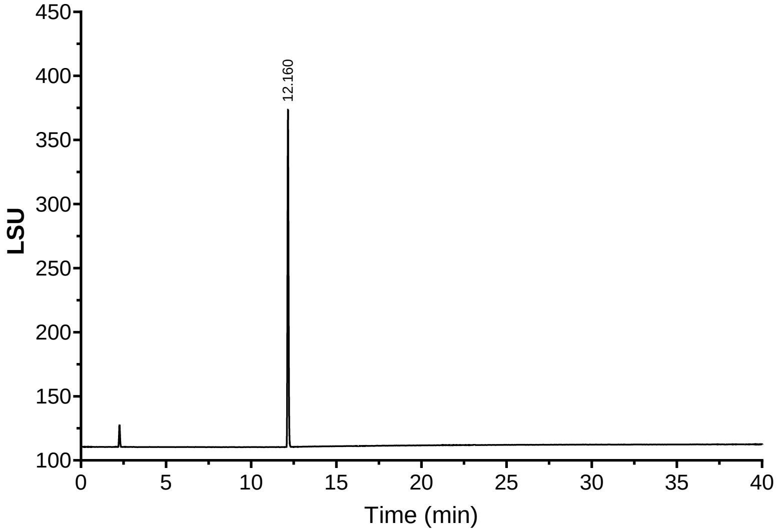

ix. Synthesis of G10.

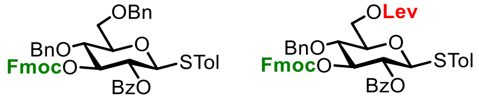

BB20 **BB22**

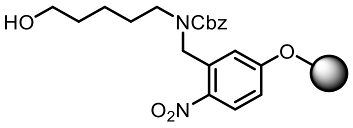

1

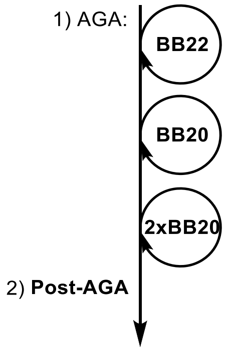

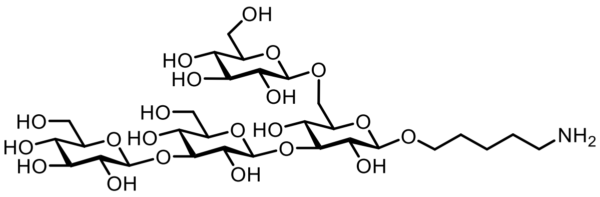

G10

Step		Modules	Notes
AGA		**A**	
	**BB22**	**B, C, D, E1**	**C:** (-20 °C for 15 min, 0 °C for 30 min)
	**BB20**	**B, C, D, E3, E1**	**C:** (-20 °C for 15 min, 0 °C for 30 min)
	**2x BB20**	**B, C, D, E1**	**C:** (-20 °C for 15 min, 0 °C for 30 min)
Post-AGA	**Methanolysis**	**F**	**F:** 18 h
	**Photocleavage**	**G**	
	**Hydrogenolysis**	**I**	**I:** 18 h (2 mL, 2:1:1 EtOAc:H_2O:*t*BuOH)
	**Purification**	**J_{1P}**	

Compound **G10** was obtained as a white solid (0.3 mg, 3% overall yield).

Analytical data for **G10**:

^{1}H NMR (400 MHz, D_2O) δ 4.69 (d, *J* = 7.9 Hz, 2H), 4.48 – 4.41 (m, 2H), 4.16 (d, *J* = 11.7 Hz, 1H), 3.86 (d, *J* = 12.2 Hz, 5H), 3.75 – 3.61 (m, 6H), 3.57 – 3.19 (m, 14H), 2.94 (t, *J* = 7.4 Hz, 2H), 1.69 – 1.54 (m, 4H), 1.40 (m, 2H).

^{13}C NMR (101 MHz, D_2O) δ 102.7, 102.4, 84.0, 75.8, 74.5, 73.2, 73.2, 72.7, 70.3, 69.5, 68.5, 68.1, 60.6, 60.5, 39.4, 27.9, 26.6, 22.1

HRMS (QToF): Calculated for $C_{29}H_{54}NO_{21}$ $[M+H]^+$ 752.3183; found 752.3188.

^{1}H NMR of G10 (400 MHz, D_2O)

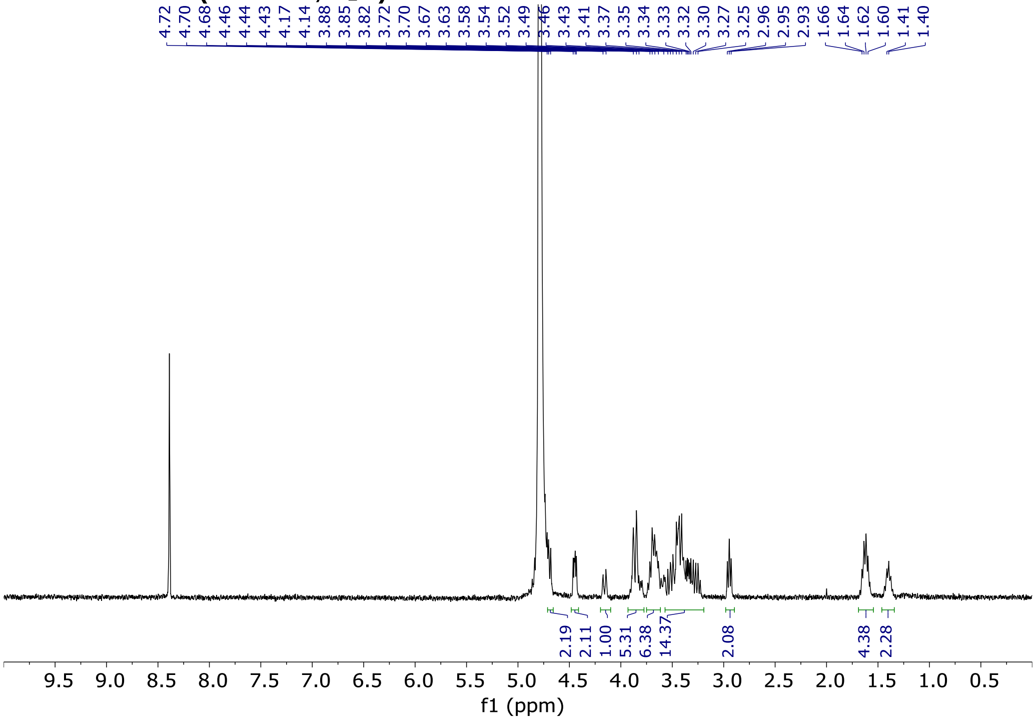

^{1}H-^{1}H COSY NMR of G10 (D_2O)

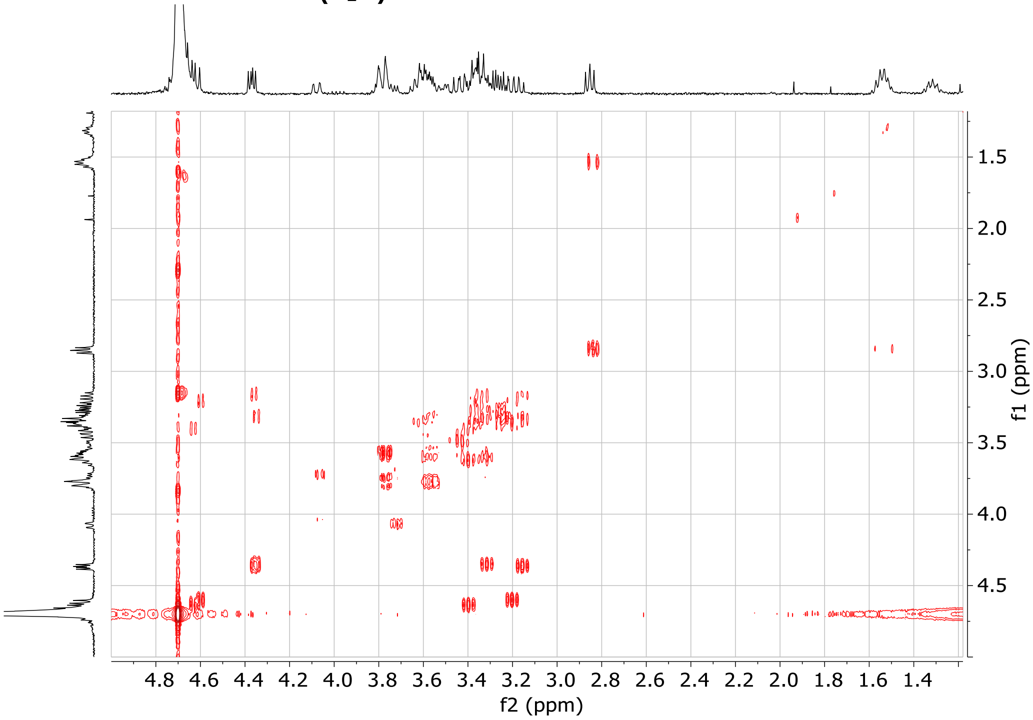

^{1}H-^{13}C HSQC NMR of G10 (D_2O)

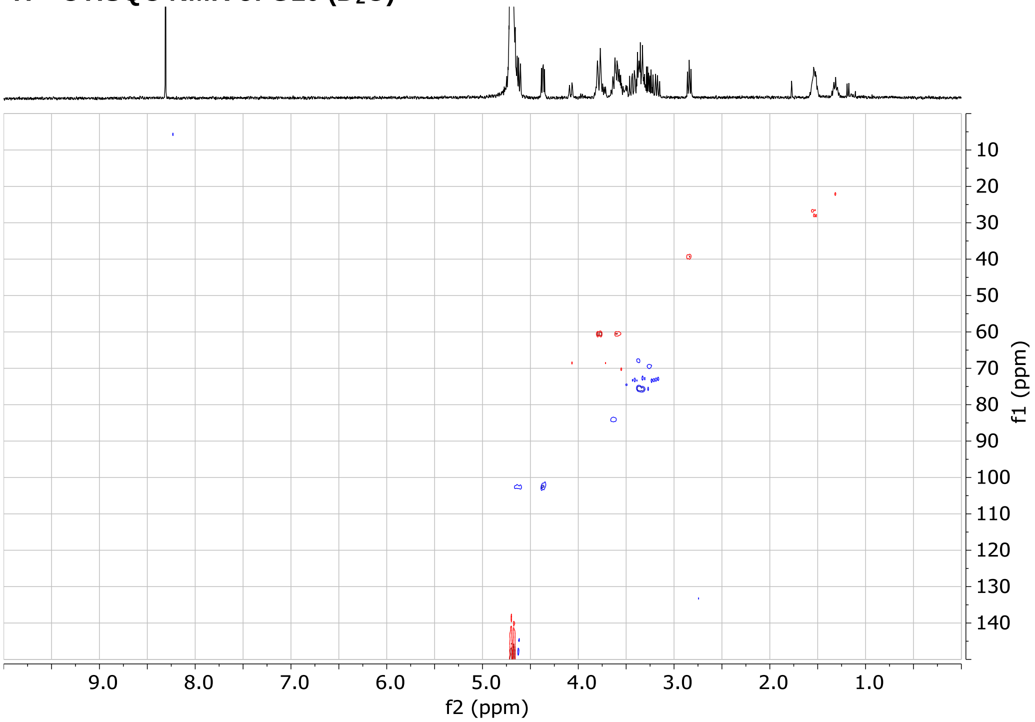

RP-HPLC of G10 (ELSD trace, Method J_{1A}, t_R= 12.28 min)

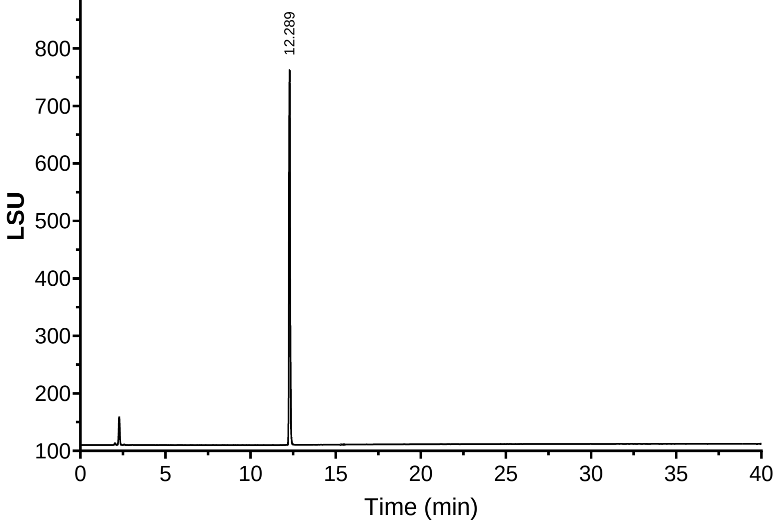

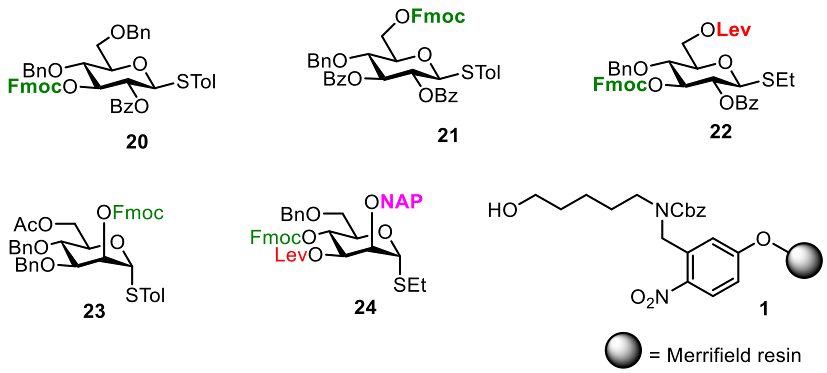

Figure S 1. Building blocks and resins utilized for AGA.

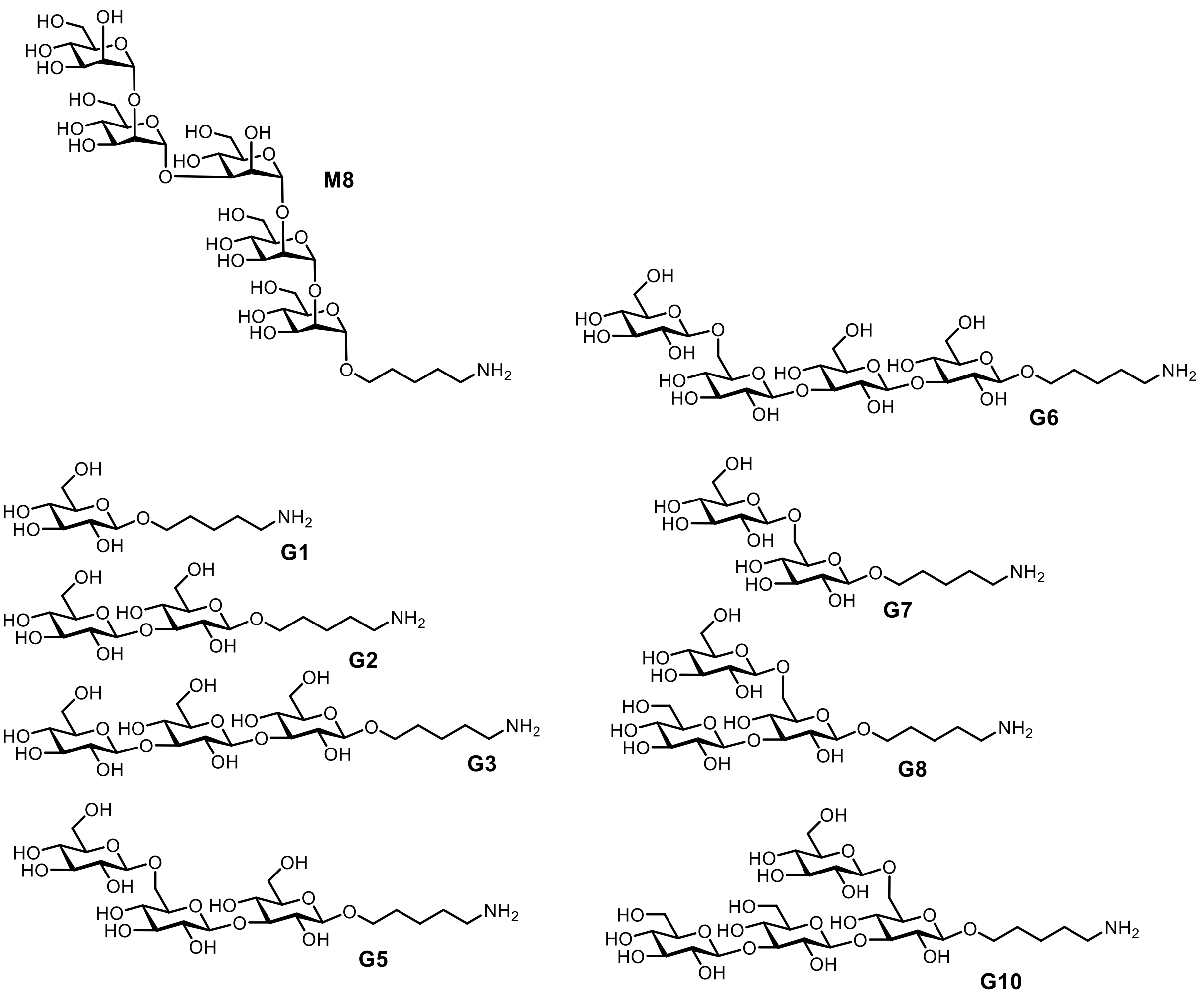

Figure S 2. Synthetized glycans.

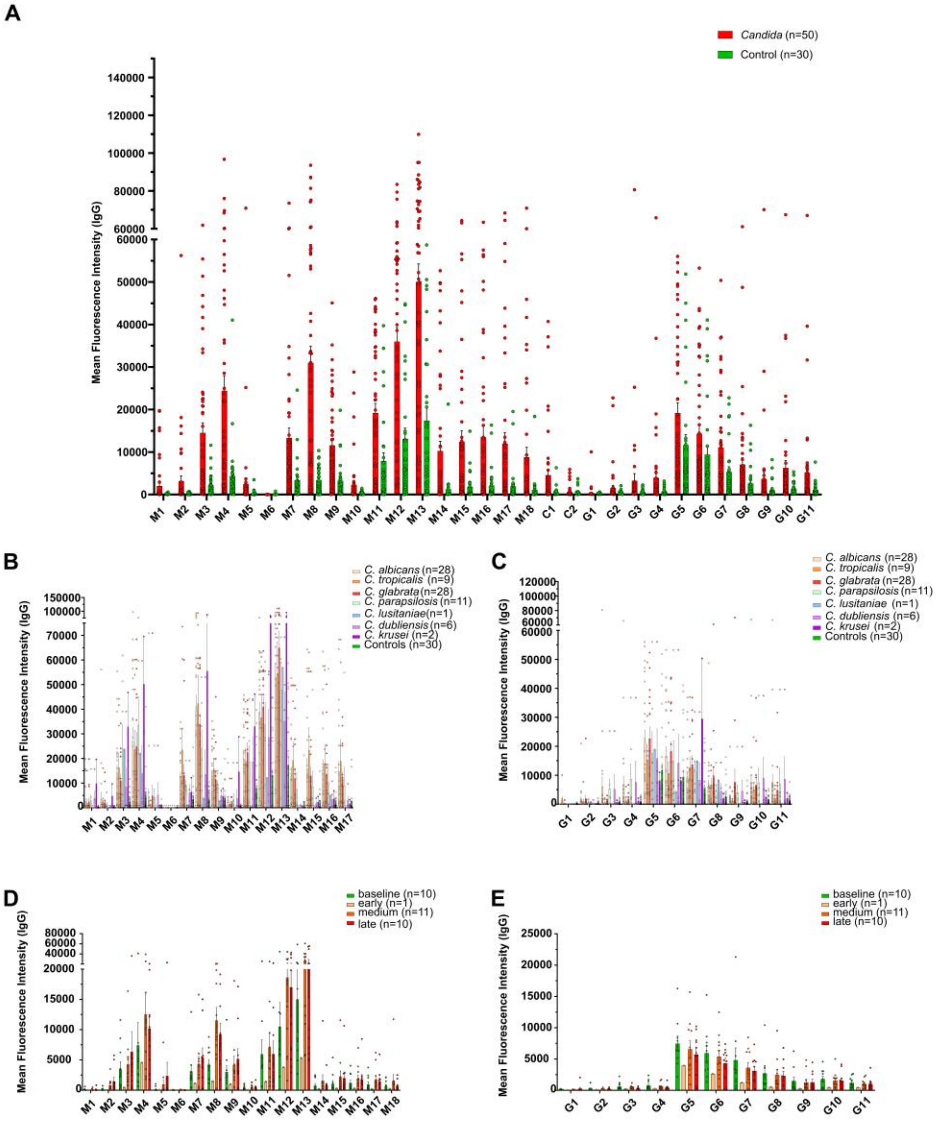

Figure S 3. Mean fluorescence intensity of IgG antibody binding to synthetic glycans in individual humans. Mean fluorescence intensity of IgG antibodies in sera derived from humans with invasive candidiasis, binding to synthetic mannans and β-glucans (A). Mean fluorescence intensity of IgG antibodies in sera derived from humans with different *Candida* spp. infections, binding to synthetic mannans (B) or β-glucans (C). Mean fluorescence intensity of IgG antibodies in sera derived from humans before (baseline) or at an early (1 day), medium (5-8 days) or late (12-16 days) timepoint after positive blood culture with invasive *Candida* spp., binding to synthetic mannans (D) or β-glucans (E). A serum dilution of 1:100 was used. Values represent mean ± SEM. Value from each sample is represented as a dot. Differences were tested for significance to healthy controls (A-C) or baseline (D-E) using multiple Mann-Whitney test with (***) $p < 0.001$, (**) $p < 0.01$ and (*) $p < 0.05$.

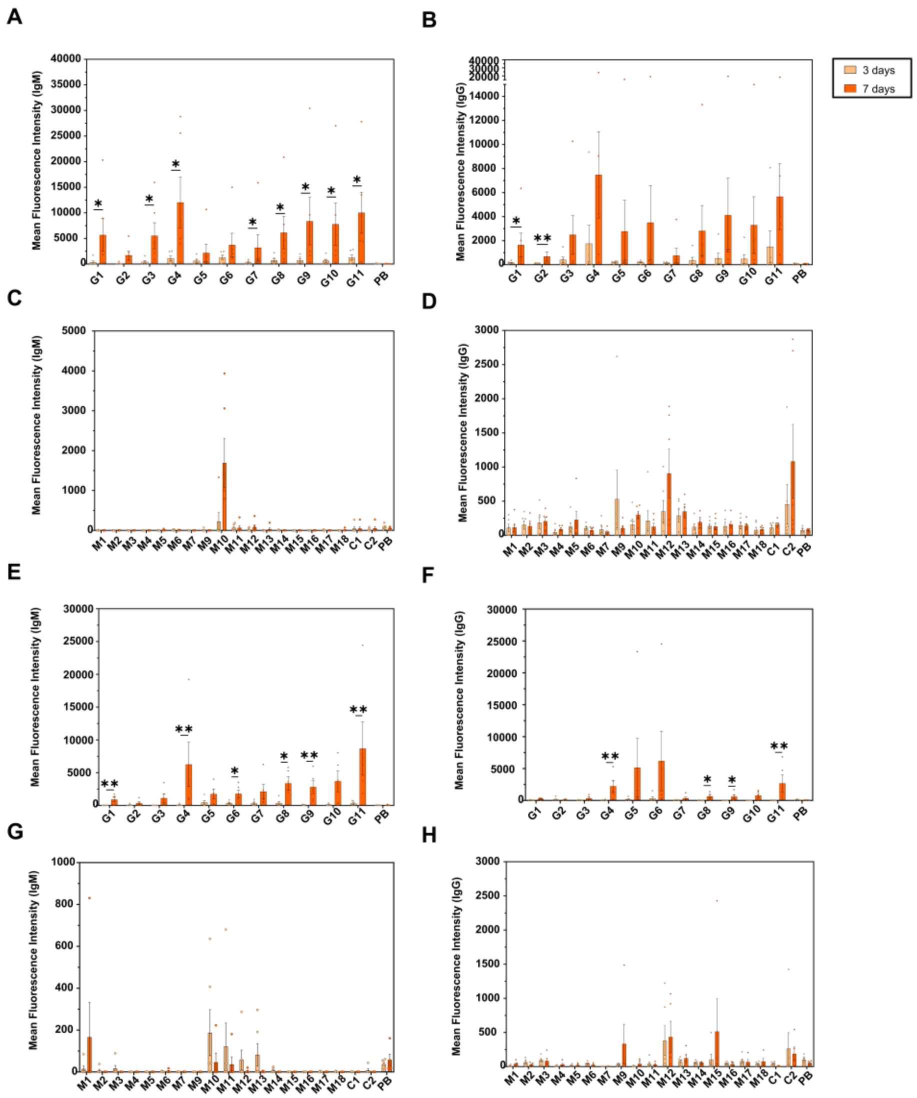

Figure S 4. Mean fluorescence intensity of IgM and IgG antibody binding to synthetic glycans in a mouse model of early invasive candidiasis. Mean fluorescence intensity of IgM antibody (A, C) and IgG antibody (B, D) binding to synthetic β-glucans (A, B) and mannans (C, D) after three days or seven days of infection with live *C. albicans* (CWZ 10061110). Mean fluorescence intensity of IgM antibody (E, G) and IgG antibody (F, H) binding to synthetic β-glucans (E, F) and mannans (G, H) after three days or seven days of infection with live *C. auris* (CWZ 10051896) (belonging to clade I). A serum dilution of 1:100 was used. Values represent mean ± SEM. Value from each sample is represented as a dot. Differences were tested for significance to healthy controls (A) or three days after infection (B-H) using multiple Mann-Whitney test with (***) p <0.001, (**) p <0.01 and (*) p <0.05.

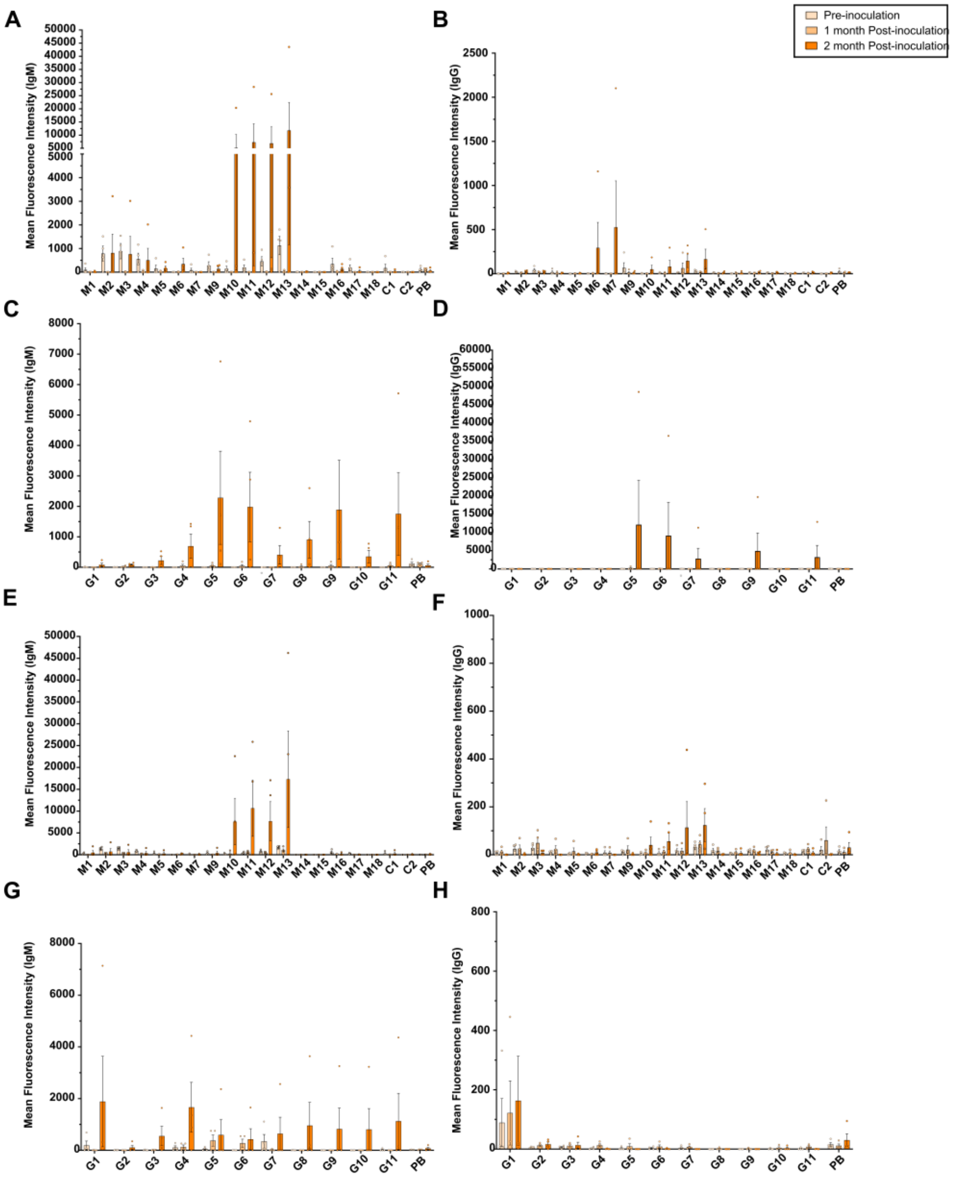

Figure S 5. Mean fluorescence intensity of IgM and IgG antibody binding to synthetic glycans in a mouse model of late invasive candidiasis. Mean fluorescence intensity of IgM antibody (A, C, E, G) and IgG antibody (B, D, F, H) binding to synthetic mannans (A, B, E, F) and *β*-glucans (C, D, G, H) after one month or two months of inoculation with killed *C. auris* NCPF13001#16 (clade 1) (A-D) or *C. auris* VPCI479/P/13 (clade 1) (E-H). A serum dilution of 1:100 was used. Values represent mean ± SEM. Value from each sample is represented as a dot. Differences were tested for significance to pre-inoculation using multiple Mann-Whitney test with (***) p <0.001, (**) p <0.01 and (*) p <0.05.

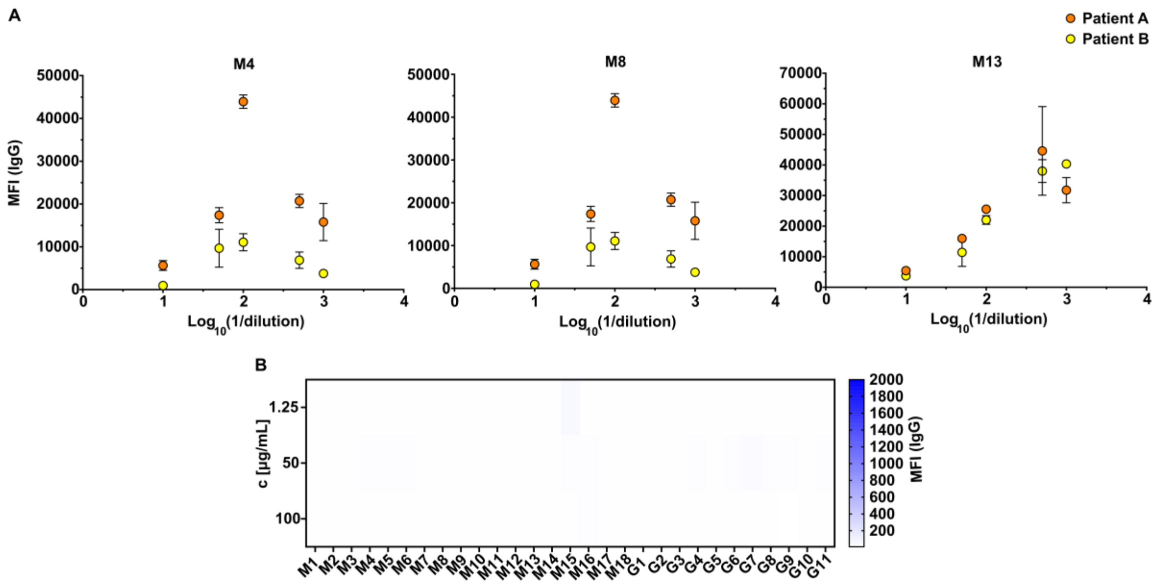

Figure S 6. Mean fluorescence intensity (MFI) of IgG antibody binding to synthetic glycans to determine optimal dilution of sera. (A) MFI of IgG antibody binding to synthetic glycans M4, M8 and M13 in sera derived from two patients at different dilutions (1:10, 1:50, 1:100, 1:500 and 1:1000). (B) MFI of control isotype IgG antibody (goat anti-human IgG Fc-AF647 (SouthernBiotech) binding to synthetic glycans at different concentrations (1.25 µg/mL, 50 µg/mL and 100 µg/mL). Values represent mean (B) ± SEM (A).

SI References

1. J. Danglad-Flores *et al.*, Optimized platform for automated glycan assembly. *Device* **2**, 100499 (2024).
2. M. Gude, J. Ryf, P. D. White, An accurate method for the quantitation of Fmoc-derivatized solid phase supports. *Letters in Peptide Science* **9**, 203-206 (2002).
3. S. Eller, M. Collot, J. Yin, H. S. Hahm, P. H. Seeberger, Automated solid-phase synthesis of chondroitin sulfate glycosaminoglycans. *Angew Chem Int Ed Engl* **52**, 5858-5861 (2013).
